# Drift, not selection, shapes toll‐like receptor variation among oceanic island populations

**DOI:** 10.1111/mec.13437

**Published:** 2015-11-24

**Authors:** Catalina Gonzalez‐Quevedo, Lewis G. Spurgin, Juan Carlos Illera, David S. Richardson

**Affiliations:** ^1^ School of Biological SciencesUniversity of East AngliaNorwich Research ParkNorwichNR4 7TJUK; ^2^ Grupo Ecología y Evolución de VertebradosInstituto de BiologíaFacultad de Ciencias Exactas y NaturalesUniversidad de AntioquiaCalle 70 No. 52‐21MedellinColombia; ^3^ Research Unit of Biodiversity (UO‐CSIC‐PA)Oviedo UniversityCampus of MieresResearch Building5th Floor. C/Gonzalo Gutiérrez Quiróss/n, 33600 MieresAsturiasSpain

**Keywords:** *Anthus berthelotii*, Berthelot's pipit, bottleneck, founder effects, genetic drift, genetic variation, selection, toll‐like receptors

## Abstract

Understanding the relative role of different evolutionary forces in shaping the level and distribution of functional genetic diversity among natural populations is a key issue in evolutionary and conservation biology. To do so accurately genetic data must be analysed in conjunction with an unambiguous understanding of the historical processes that have acted upon the populations. Here, we focused on diversity at toll‐like receptor (TLR) loci, which play a key role in the vertebrate innate immune system and, therefore, are expected to be under pathogen‐mediated selection. We assessed TLR variation within and among 13 island populations (grouped into three archipelagos) of Berthelot's pipit, *Anthus berthelotii*, for which detailed population history has previously been ascertained. We also compared the variation observed with that found in its widespread sister species, the tawny pipit, *Anthus campestris*. We found strong evidence for positive selection at specific codons in TLR1LA, TLR3 and TLR4. Despite this, we found that at the allele frequency level, demographic history has played the major role in shaping patterns of TLR variation in Berthelot's pipit. Levels of diversity and differentiation within and across archipelagos at all TLR loci corresponded very closely with neutral microsatellite variation and with the severity of the bottlenecks that occurred during colonization. Our study shows that despite the importance of TLRs in combating pathogens, demography can be the main driver of immune gene variation within and across populations, resulting in patterns of functional variation that can persist over evolutionary timescales.

## Introduction

Genetic variation provides the fundamental substrate for evolution. Consequently, understanding the levels and distribution of functional genetic diversity among individuals and populations, and what forces drive these patterns, is a central component of evolutionary biology. Given that genetic variation is critical to the adaptive potential of populations and species, this understanding also has important implications for conservation (Frankham *et al*. 1999).

Population bottlenecks result in losses of functional genetic diversity (Cabe 1998; Gautschi *et al*. 2002; Sutton *et al*. 2013) and are a key force in shaping the future evolution and persistence of populations (Frankham 1996; England *et al*. 2003). When populations undergo bottlenecks, genetic drift is usually the dominant force, reducing genetic diversity within populations and driving differentiation across them (Hartl & Clark 2007). However, if selection acts on a specific region of the genome it can either counteract, or alternatively, reinforce the effects of drift depending on the type of selection operating (Aguilar *et al*. 2004; Miller & Lambert 2004). Under balancing selection, genetic diversity will be maintained within the bottlenecked populations, reducing the amount of differentiation one might expect under drift alone (Hedrick & Thomson 1983; Hughes & Nei 1988), at least at the specific loci involved. Alternatively, under purifying or constant directional selection, genetic diversity will be reduced and the effects of drift, and resulting population differentiation, will be reinforced (Jiggins & Hurst 2003; Winternitz & Wares 2013).

When investigating genetic variation, loci involved in the immune system are of particular interest, not least because of their obvious importance for individual and population survival (reviewed in Sommer 2005; Acevedo‐Whitehouse & Cunningham 2006), but also because they are expected to be under strong and direct selection from pathogens (Trowsdale & Parham 2004; Ekblom *et al*. 2010). Over the last few decades, genes of the major histocompatibility complex (MHC), which code for receptors central to the acquired immune system, have been the focus of studies into functional genetic diversity and pathogen‐mediated selection among wild populations (reviewed in Piertney & Oliver 2006; Spurgin & Richardson 2010). However, the MHC is highly complex in terms of its structure and evolution, making it extremely difficult to disentangle the interacting ecological and evolutionary forces driving variation in natural populations (Spurgin & Richardson 2010). Only recently has attention spread to investigating other important (and potentially more tractable) immune genes (Acevedo‐Whitehouse & Cunningham 2006; Jensen *et al*. 2008; Grueber *et al*. 2012; Turner *et al*. 2012), and recent studies have started to investigate the evolutionary forces shaping variation at these genes (Bollmer *et al*. 2011; Tschirren *et al*. 2012; Grueber *et al*. 2013, 2015; Hartmann *et al*. 2014; Quéméré *et al*. 2015).

Toll‐like receptors (TLRs) are essential components of the innate immune response (Roach *et al*. 2005). They bind pathogen‐associated molecular patterns (PAMPs), thus triggering an intracellular signalling cascade that results in an inflammatory response and activation of macrophages, which attack the infection (Belvin & Anderson 1996; Akira 2003). Vertebrate TLRs are divided into six families that vary in the type of PAMPs they recognize (Roach *et al*. 2005). For example, the TLR1 family bind bacterial lipoproteins (Takeuchi *et al*. 2002), TLR3 binds viral RNA (Yoneyama & Fujita 2010), TLR21—an avian‐specific gene—binds bacterial DNA motifs (Keestra *et al*. 2010), and TLR4 binds bacterial lipopolysaccharide (Poltorak *et al*. 1998). Recent studies of the loci that encode these molecules have shown evidence of positive selection in TLR loci in a range of organisms including birds (Downing *et al*. 2010; Alcaide & Edwards 2011; Grueber *et al*. 2014), fish (Chen *et al*. 2008) and mammals (Nakajima *et al*. 2008; Areal *et al*. 2011; Tschirren *et al*. 2011; Quéméré *et al*. 2015). Most of the sites identified as being under positive selection in these studies are located in the TLR extracellular domain which directly binds the PAMPs (reviewed in Mikami *et al*. 2012), and specific polymorphisms within TLR loci have been associated with differential disease resistance (Schröder & Schumann 2005; Misch & Hawn 2008; Basu *et al*. 2010). These data thus support the idea that pathogen‐mediated selection plays a role in determining patterns of variation at TLR loci.

While the relationship between individual‐level TLR variation and an organism's ability to resist infection is becoming clearer, how different evolutionary forces interact to shape TLR variation at the level of populations and species remains poorly understood. Studying population‐level variation at these critical genes, especially in bottlenecked populations and/or endangered species, is important from both an evolutionary and conservation perspective (Grueber *et al*. 2012; Hartmann *et al*. 2014). Although there have been studies of TLR variation across species (Nakajima *et al*. 2008; Wlasiuk & Nachman 2010; Areal *et al*. 2011; Mikami *et al*. 2012), few studies have investigated TLR variation within bottlenecked populations (Grueber *et al*. 2012; Hartmann *et al*. 2014), or how TLR variation is distributed across multiple natural populations of a single species (Tschirren *et al*. 2012; Quéméré *et al*. 2015). Furthermore, most of these studies focus on the effects of recent demographic events and do not tell us about what determines among population patterns of TLR variation over longer timescales.

**Figure 1:**
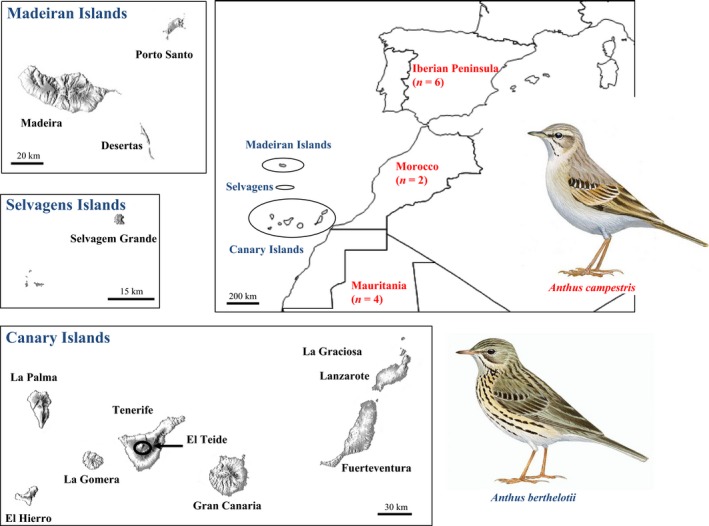
Distribution of Berthelot's pipit, *Anthus berthelotii*, across the Islands of the Macaronesian archipelagos and the populations of tawny pipits, *Anthus campestris*, used in this study. The identity of each island and the sample sizes for tawny pipits are shown. Sample sizes for Berthelot's pipit from each island are given in Table S1 (Supporting information).

Berthelot's pipit, *Anthus berthelotii*, is a small, insectivorous passerine endemic to the North Atlantic archipelagos of Madeira, Selvagens and the Canary Islands where it occurs in relatively isolated populations of varying size (Cramp & Perrins 1977–1994; Fig. 1). The species is thought to have colonized the Macaronesian archipelagos from Africa, where its closest relative the tawny pipit (*Anthus campestris*) inhabits, during the Pleistocene (Voelker 1999). Previous work on Berthelot's pipit has provided evidence of how population bottlenecks of differing severity, which occurred during the colonization of each archipelago, have been dominant in shaping neutral variation across the populations (Illera *et al*. 2007; Spurgin *et al*. 2014). These studies have shown that genetic variation at microsatellites is highest in the Canary Islands and lowest in the more recently colonized Madeira and Selvagens archipelagos (*c*. 8500 years ago). We have also shown that these populations are exposed to spatially varying pathogen pressures that are consistent over time (Spurgin *et al*. 2012) and that MHC genes are rapidly evolving across the populations as a result of gene conversion and positive selection (Spurgin *et al*. 2011). Thus, this species provides an ideal study system in which to investigate the roles of selection vs. drift in determining patterns of functional genetic diversity in fragmented bottlenecked populations. With that as our overall aim, here we characterized variation at five of the ten known avian TLR loci in Berthelot's pipit. Specifically we (i) characterized genetic variation at TLR loci in Berthelot's pipit and compared it to that found in other bird species, including its sister species, the tawny pipit, (ii) investigated how TLR genetic diversity is distributed within and among 13 Berthelot's pipit populations and (iii) assessed the relative roles of selection and drift in shaping the patterns of variation we observed in these important immune loci.

## Methods

### Study populations and sampling

We screened individuals from all islands sampled as part of earlier studies (Illera *et al*. 2007; Spurgin *et al*. 2012; Fig. 1, Table S1, Supporting information). A 13th population was sampled from El Teide, a volcano situated at the centre of Tenerife rising 3718 m above sea level (a.s.l.), due to its geographically isolated position from the others. The El Teide population exists at >2000 m a.s.l. across an alpine plateau (*c*. 190 km^2^), separated from the rest of the island population by its altitude and a ring of pine forest that extends from approximately 1600–2000 m a.s.l., which is not suitable habitat for pipits (Illera *et al*. 2007). Overall, it is plausible to consider El Teide a different population. Birds were captured using clap nets baited with *Tenebrio molitor* larvae. Blood samples were collected by brachial venipuncture, diluted in 800 μL absolute ethanol in screw‐cap microcentrifuge tubes and stored at room temperature. We also obtained blood samples from 12 tawny pipits (two from Morocco, four from Mauritania and six from the Iberian Peninsula), captured using the same methods as for Berthelot's pipits.

### Estimation of divergence time between Berthelot's and tawny pipits

Berthelot's and tawny pipits shared some haplotypes at TLRs (see Results). To assess whether this was the result of limited time for lineage sorting to occur since the two species split or whether it was evidence of trans‐species polymorphism we estimated time of divergence between the two species. Previously, the timing of divergence between Berthelot's and tawny pipits had been estimated using DNA from a tawny pipit from Denmark (Voelker 1999). However, a phylogenetic analysis suggests that the migratory behaviour of tawny pipits evolved after the split between Berthelot's and tawny pipits (Outlaw & Voelker 2006). Thus Berthelot's pipits likely evolved from an ancestral sedentary population that existed near to the Canary Islands and, therefore, the divergence time might have been overestimated. We therefore re‐estimated divergence time based on mitochondrial gene cytochrome oxidase subunit I (COI) sequence data, using tawny pipits from the Iberian Peninsula and northern Africa (Mauritania and Morocco, Appendix S1, Supporting information).

### TLR genotyping

DNA was extracted using a salt extraction method following Richardson *et al*. (2001). In both Berthelot's and tawny pipits we amplified TLR1LA, TLR1LB and TLR3 using the primers published by Alcaide & Edwards (2011), and TLR4 and TLR21 with the primers published by Grueber *et al*. (2012). These primers amplify fragments (mean size = 862 bp, ranging from 622 to 1041 bp) of the TLRs extracellular domains, the regions directly involved in pathogen recognition. We were unable to successfully amplify a further four TLRs (TLR2A, TLR2B, TLR5 and TLR15) using either the primers available in Alcaide & Edwards (2011) or Grueber *et al*. (2012). Polymerase chain reactions (PCR) were performed in a Tetrad thermocycler (MJ Research) using the following profile: initial denaturing at 94 °C for 4 min, followed by 35 cycles of denaturing at 94 °C for 40 s, annealing at 60 °C (TLR1LA, TLR3, TLR4 and TLR21) or 64 °C (TLR1LB) for 40 s and extension at 72 °C for 80 s; a final extension step was performed at 72 °C for 10 min. Reactions were conducted in 10 μL volumes using the TopTaq polymerase master mix kit (Qiagen), 0.5 μm of each primer and *c*. 30 ng of DNA. Amplified fragments were visualized on 2% agarose gels stained with ethidium bromide and purified using a mixture of recombinant alkaline phosphatase and exonuclease I, incubating at 37 °C for 30 min, followed by enzyme inactivation at 95 °C for 5 min. Amplified fragments were sequenced using the BigDye terminator kit (Applied Biosystems) using the following thermal profile: 96 °C for 2 min, followed by 25 cycles of 96 °C for 10 s, 50 °C for 5 s and 60 °C for 4 min. Products were visualized using an ABI genetic analyzer (Applied Biosystems). Sequences were aligned and edited in bioedit 7.0.9.0 (Hall 1999) and single nucleotide polymorphisms (SNPs) were detected by visually examining chromatograms. The International Union of Pure and Applied Chemistry code for degenerate nucleotides was used for heterozygous positions. Individual haplotypes were resolved using the PHASE algorithm (Stephens *et al*. 2001) implemented in dnasp 5.10.01 (Librado & Rozas 2009). All haplotypes were confirmed by repeat PCR and sequencing from at least two samples.

### Analyses

All analyses were carried out in r version 3.0.2 (R Development Core Team 2011), unless stated otherwise. To determine the relationship between alleles from each TLR across different bird species, we constructed maximum‐likelihood trees for each locus using 1000 bootstrap replications and the general time reversible substitution model using mega 6 (Tamura *et al*. 2013). These trees included sequences from the two pipit species obtained in this study and three species obtained from GenBank: the house finch, *Carpodacus mexicanus,* lesser kestrel, *Falco naumanni* (Alcaide & Edwards 2011), and the New Zealand robin, *Petroica australis rakiura* (Grueber *et al*. 2012). To further visualize relationships between TLR genes in Berthelot's and tawny pipits, we built haplotype networks using hapstar 0.5 (Teacher & Griffiths 2011).

The ratio (ω) of nonsynonymous to synonymous substitutions per site (d*N*/d*S*) was calculated in mega 6 (Tamura *et al*. 2013) for each locus using the haplotype sequences identified in Berthelot's and tawny pipits. Significance of the relationship between d*N* and d*S* (d*N* < d*S*, d*N* > d*S*) was tested with 10 000 bootstrap replications. This whole haplotype method gives an indication of selection averaged over all sites in the sequence but requires a strong signal to detect selection (Pond & Frost 2005b). To explore whether specific codons, rather than the entire sequence, were under positive selection, two codon‐based methods were used: fast unbiased Bayesian approximation (FUBAR) (Murrell *et al*. 2013) and the mixed‐effects model of evolution (MEME; Murrell *et al*. 2012). These approaches are the most up‐to‐date for detecting pervasive (FUBAR) and episodic (MEME) selection at individual codons and are intended to supersede older methods (e.g. REL, SLAC, FEL) due to their improved power and efficiency (Murrell *et al*. 2012, 2013). Sites with posterior probabilities >0.9 for FUBAR, and *P* values <0.1 for MEME were considered to support positive selection. Prior to running analyses, the best fitting nucleotide substitution model was determined for each locus using a model selection approach (Pond & Frost 2005a). These tests were all run in Datamonkey (http://datamonkey.org, Pond & Frost 2005a) with sequences from the two pipit species. Gene conversion and recombination were estimated for each locus using methods described by Betrán *et al*. (1997) and Hudson (2007), respectively, implemented in dnasp 5.10.01 (Librado & Rozas 2009).

To best visualize genetic diversity among Berthelot's pipit populations, islands were pooled into archipelagos. This is in accordance with findings from previous research, which showed that most structure is at the archipelago level (Spurgin *et al*. 2014), and analysis of structure at TLRs confirmed the validity of this approach (see Results). We calculated measures of TLR nucleotide diversity in mega 6 (Tamura *et al*. 2013) and tested for differences between the two pipit species, and among archipelagos for Berthelot's pipit, using randomization tests (Manly 2007). Tests of Hardy–Weinberg equilibrium were carried out using the web version of genepop (http://genepop.curtin.edu.au/, Raymond & Rousset 1995). Nucleotide diversity comparisons involving Selvagem Grande were not possible because of the low number of haplotypes detected in this population (see Results). In addition to nucleotide diversity, for each TLR locus and population, we calculated the number of alleles and expected heterozygosity using custom r scripts. We also calculated allelic richness after correcting for differences in sample size between the archipelagos (as a result of there being different numbers of populations per archipelago) using the software hp‐rare 1.0 (Kalinowski 2005).

We limited analyses of genetic differentiation to the Berthelot's pipit, due to the low sample size available for tawny pipits. A previous study of Berthelot's pipits identified a pattern of ‘isolation by colonization’, whereby neutral genetic structure (at microsatellites) among populations was largely the product of the relative bottleneck severity of populations (hereafter ‘bottleneck distance’; Spurgin *et al*. 2014). Here, we assessed whether TLR genetic structure among islands was consistent with this pattern. As an index of bottleneck distance, we used a modified version of Garza & Williamson's (2001) *M* ratio, developed to reflect the relative bottleneck severity of pairs of islands based on microsatellite variation (Spurgin *et al*. 2014). We calculated pairwise *F*_ST_ between all islands in arlequin v3.5 (Excoffier & Lischer 2010). For every microsatellite and TLR locus, we tested whether pairwise *F*_ST_ was related to bottleneck distance with a Mantel test, implemented in the r package ecodist (Goslee & Urban 2007). We then compared the distribution of correlation coefficients from the Mantel tests for microsatellite and TLR loci. A strong correlation between the pairwise *F*_ST_ values based on TLR variation and bottleneck distance would indicate that the distribution of TLR variation among populations follows the same pattern of isolation by colonization as the microsatellites. If no correlation is found, the patterns of genetic structure may be the product of population specific patterns of selection.

## Results

### Divergence time between Berthelot's and tawny pipits

We found eight and three variable sites within the tawny and Berthelot's pipit COI haplotypes, respectively. The mean estimated time to most recent common ancestor of all Berthelot's and tawny pipit haplotypes was 2.34 Myr (95% HPD = 1.57–3.13 Myr).

### TLR variation within and across species

We initially screened variation at each TLR locus in 5 individuals per population. The highest levels of functional variation were observed at TLR3 and TLR4, so an additional 25 individuals per population were screened at these loci to ensure we detected all functional variants. The remaining loci were genotyped at an additional 5 individuals per population (see Table 1 for final sample sizes). Twelve tawny pipits were all screened at all five TLR loci. A summary of variation across the five TLR loci is given in Table S2 (Supporting information). No SNPs resulted in frame shifts or stop codons. A total of 41 alleles were identified in Berthelot's pipits (Table S3, Supporting information), of which 24 occurred as homozygotes and the remaining 17 were inferred using PHASE. We found 5–10 haplotypes per locus, which translated to between 3 and 6 different amino acid variants at each locus (Table 1). In tawny pipits, we identified a total of 62 alleles (Table S3, Supporting information), with between 7–18 haplotypes and 5–11 amino acid variants per locus (Table 1).

**Table 1:** Polymorphism at five toll‐like receptor loci in the three Berthelot's pipit (*Anthus berthelotii*) archipelago populations (CI = Canary Islands, M = Madeira, S = Selvagem Grande) and the sister species, the tawny pipit (*Anthus campestris*)

Locus	Population (*N*)	Sites^a^	Alleles^b^	π (SD)^c^	AA^d^	AR^e^	*H*_d_ f	*H*_o_ g	*H*_e_ h	HWE *P* i
TLR1LA	*A. berthelotii* (146)	8	10	24.2 (3.3)	5					
	CI (100)	8	10	24.2 (3.3)	5	5.15	0.73	0.65	0.72	0.379
	S (14)	1	2	9.9 (4.9)	1	2.00	0.48	0.28	0.46	0.240
	M (32)	3	4	16.5 (4.4)	2	3.94	0.62	0.53	0.58	0.075
	*A. campestris* (12)	21	15	38.4 (5.7)	9	9.27	0.89	0.92	0.87	0.881
TLR1LB	*A. berthelotii* (145)	9	10	23.1 (2.8)	5					
	CI (99)	6	7	21.5 (3.6)	3	3.37	0.50	0.59	0.50	0.134
	S (14)	1	2	10.3 (5.1)	2	2.00	0.45	0.07	0.44	0.003
	M (32)	6	7	19.5 (3.1)	2	5.57	0.75	0.69	0.74	0.713
	*A. campestris* (12)	18	10	44.8 (5.8)	5	5.50	0.62	0.75	0.75	1.000
TLR3	*A. berthelotii* (371)	8	9	17.1 (1.8)	6					
	CI (266)	7	8	16.8 (2.0)	5	6.56	0.53	0.49	0.58	0.114
	S (25)	1	2	9.6 (4.8)	1	2.00	0.39	0.52	0.38	0.144
	M (80)	3	4	14.4 (3.5)	2	4.00	0.70	0.74	0.69	0.942
	*A. campestris* (12)	15	12	30.6 (4.0)	8	7.76	0.76	1.00	0.83	1.000
TLR4	*A. berthelotii* (372)	5	7	29.5 (4.5)	6					
	CI (266)	4	5	25.8 (5.6)	5	4.22	0.58	0.52	0.58	0.027
	S (23)	0	1	0.0 (0.0)	1	1.00	0.00	0.00	0.00	N/A
	M (83)	4	4	30.3 (8.2)	4	4.00	0.74	0.75	0.74	0.415
	*A. campestris* (12)	22	18	73.1 (7.4)	11	10.24	0.96	0.83	0.93	0.353
TLR21	*A. berthelotii* (144)	4	5	28.9 (6.2)	3					
	CI (97)	3	4	28.9 (6.2)	3	2.79	0.53	0.49	0.52	0.042
	S (17)	1	2	16.1 (8.0)	1	2.00	0.40	0.41	0.39	1.000
	M (30)	1	2	16.1 (8.0)	1	2.00	0.44	0.36	0.43	0.414
	*A. campestris* (12)	6	7	32.2 (5.0)	5	4.50	0.66	0.75	0.66	0.981

Significant deviations from Hardy–Weinberg equilibrium are underlined.

a Number of polymorphic sites.

b Number of alleles.

c Nucleotide diversity × 10^4^ (Standard deviation).

d Number of amino acid variants.

e Allelic richness corrected for sampling difference.

f Haplotype diversity.

g Observed heterozygosity.

h Expected heterozygosity.

i *P*‐value of the Hardy–Weinberg equilibrium exact test with 1 million Markov chain steps.

The maximum‐likelihood phylogenies consistently show that alleles of each TLR locus cluster by species, except for the pipit species, which are paraphyletic (Figs S1–S5, Supporting information). Haplotype networks (Figs S6–S10, Supporting information) show that the two pipit species are very closely related at all TLRs and that they share haplotypes at TLR1LA and TLR3 (one shared allele each, Figs S6 and S8, Supporting information). In Berthelot's pipit, most haplotypes are separated from one another by a single point mutation. Haplotype networks of TLR1LB, TLR3 and TLR4 (Figs S7–S9, Supporting information) show a star‐like pattern, where most haplotypes are connected to one central haplotype present in all three archipelagos. The networks of TLR1LA and TLR21 haplotypes show chain‐like patterns in Berthelot's pipits (Figs S6 and S10, Supporting information). We found no evidence for gene conversion or recombination among the TLR loci (dnasp; Number of gene conversion tracts = 0). At the haplotype level, we found evidence for purifying selection at all loci, with ω values ranging from 0.13 to 0.71 in Berthelot's pipit and from 0.18 to 0.39 when including sequences from tawny pipit (Table S4, Supporting information). The FUBAR method for detecting pervasive, codon‐specific selection identified three sites under positive selection in TLR1LA, and two sites under positive selection in both TLR3 and TLR4 (Table S5, Supporting information). The MEME method for detecting episodic selection identified four of the seven sites identified by FUBAR, plus an extra nine sites under positive selection in TLR3 (Table S5, Supporting information).

**Figure 2:**
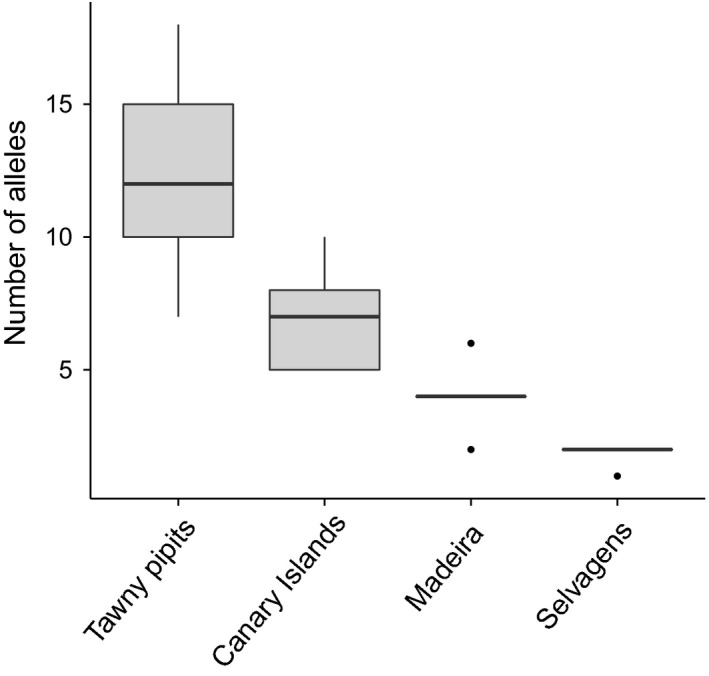
Distribution of allelic richness of five toll‐like receptor loci (after accounting for differences in sample size) across the three archipelago populations of Berthelot's pipit, *Anthus berthelotii* (Canary Islands, Madeira and Selvagens), and of tawny pipits, *Anthus campestris*. Centre lines show the medians. Box limits indicate the 25th and 75th percentiles; whiskers extend 1.5 times the interquartile range from the 25th and 75th percentiles, and outliers are represented by dots.

Genetic structure across TLR loci was very low within (mean pairwise *F*_ST_ = 0.006), but not across (mean pairwise *F*_ST_ = 0.15) archipelagos, so island populations could be pooled into archipelagos for comparisons of polymorphism. All TLR loci were polymorphic in all archipelagos, except TLR4 in the Selvagem population. Significant deviations from Hardy–Weinberg equilibrium were observed for TLR1LB in Selvagem, and TLR4 and TLR21 in the Canary Islands. In all instances a deficit of heterozygotes was found (Table 1). All measures of genetic diversity showed the same pattern, with highest levels of variation in tawny pipits, followed by Berthelot's pipit populations on the Canary Islands, then Madeira and then Selvagens, respectively (Fig. 2; Table 1). Importantly, these patterns still held when allelic richness was rarefacted, to control for sampling differences across archipelagos (Table 1). Of the Berthelot's pipit populations, the Canary Islands had the most private alleles across all loci (20), while Madeira had six and Selvagem Grande had none.

**Figure 3:**
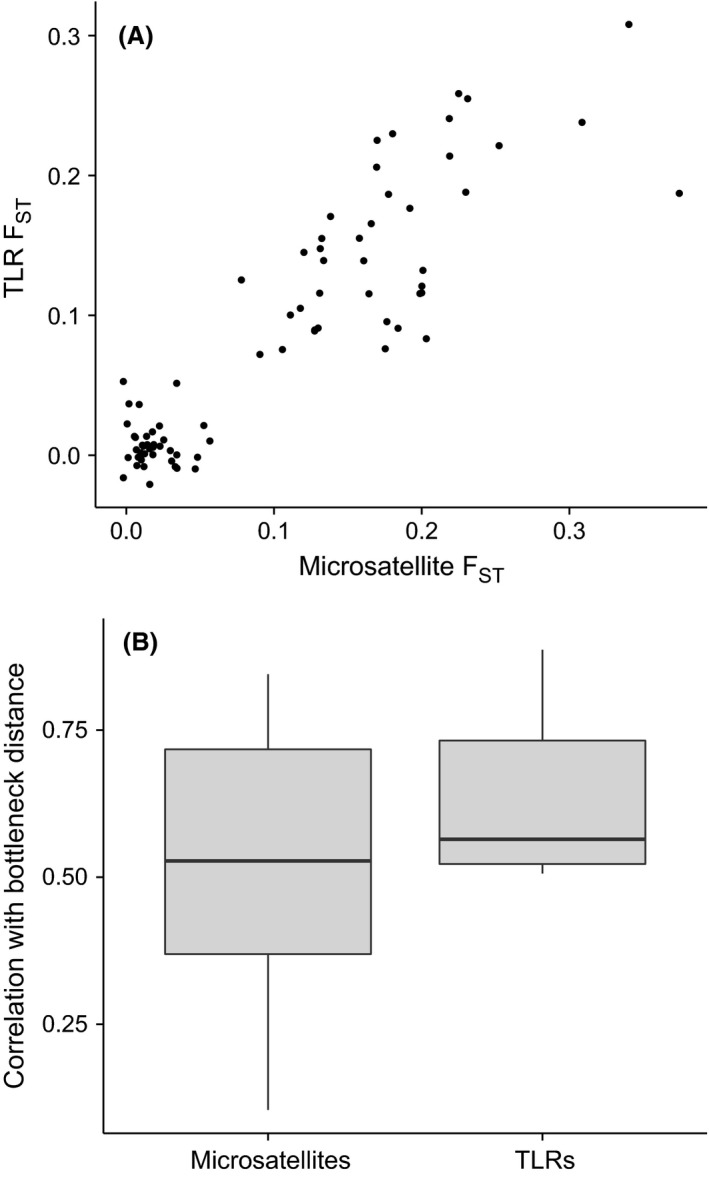
(A) Relationship between pairwise population structure at toll‐like receptor (TLR) and microsatellite loci among Berthelot's pipit (*Anthus berthelotii*) populations. (B) Distribution of Mantel correlation coefficients between pairwise *F*_ST_—for individual microsatellite and TLR loci and pairwise bottleneck distance.

**Figure 4:**
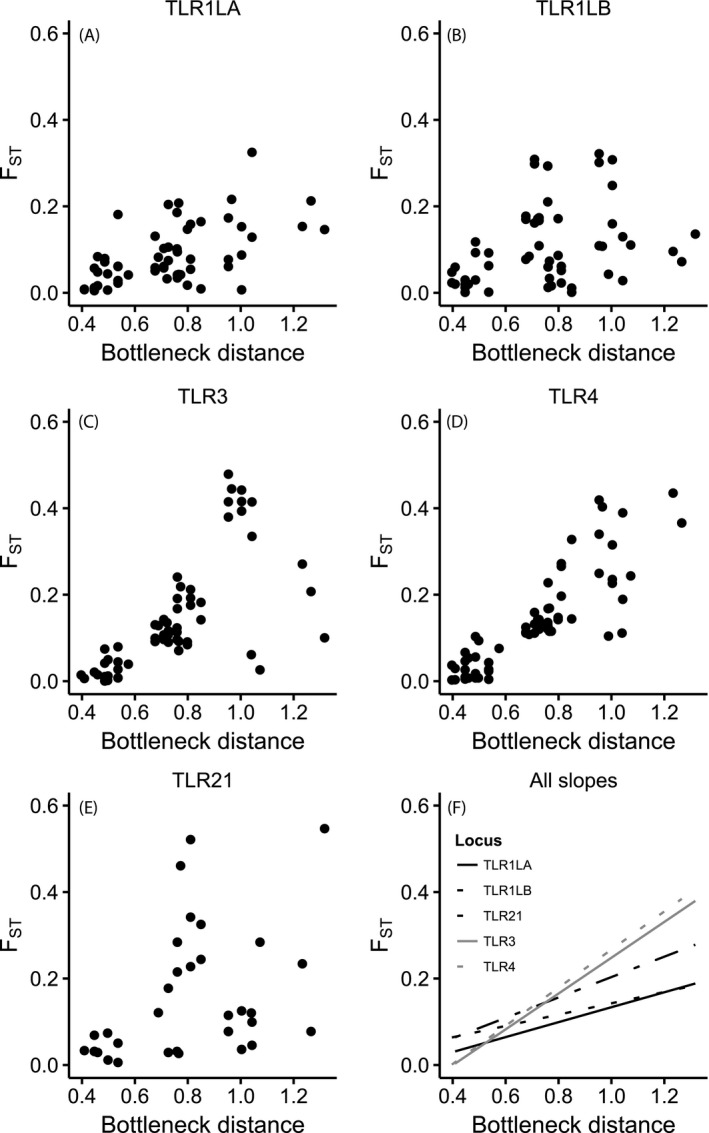
Pairwise *F*_ST_ for each of five toll‐like receptor (TLR) loci in relation to pairwise bottleneck distance between the 13 populations of Berthelot's pipit, *Anthus berthelotii*. (A–E) Scatter plot of pairwise *F*_ST_ in relation to pairwise bottleneck distance; (F) lines fit to the Mantel correlation coefficient between pairwise *F*_ST_ and pairwise bottleneck distance of the five TLRs shown in (A–E).

To assess whether neutral processes govern patterns of TLR variation among islands, we compared pairwise structure at TLRs to that at microsatellites, and with bottleneck distance. Pairwise structure averaged over TLR loci was strongly positively related to both of these variables (Mantels tests: microsatellites *r* = 0.89, *P *<* *0.001; bottleneck distance *r* = 0.88, *P *<* *0.001; Fig. 3A). Mantel *r* values from relationships between pairwise structure at individual TLR genes and bottleneck distance fell within the upper range of values obtained from Mantel tests between individual microsatellites and bottleneck distance (Fig. 3B). Indeed, we found a strong and significant positive relationship between island pairwise *F*_ST_ and bottleneck distance for TLR1LA (*r* = 0.56, *P *=* *0.003), TLR1LB (*r* = 0.51, *P *=* *0.012), TLR3 (*r* = 0.73, *P *<* *0.001) and TLR4 (*r* = 0.89, *P *<* *0.001), and a marginally nonsignificant positive relationship for TLR21 (*r* = 0.52, *P *=* *0.06) (Fig. 4).

## Discussion

Here, we show that Berthelot's pipits have reduced TLR variation compared to tawny pipits, most likely due to founder effects associated with the colonization of Macaronesia by Berthelot's pipits. In addition, in Berthelot's pipits variation at these TLR loci was further reduced as a result of the bottlenecks experienced during the recent colonization events to the Madeiran and Selvagens archipelagos from the Canaries. Importantly, despite evidence of historical selection at some TLR loci, we found that levels of population differentiation observed at the TLR loci in Berthelot's pipits reflect the bottleneck history of this species, mirroring a pattern previously found with microsatellites (Spurgin *et al*. 2014). We discuss each of these findings below.

Our phylogenetic trees—including sequences from Berthelot's pipit, tawny pipit, house finch, New Zealand robin and lesser kestrel—indicate that within each TLR locus, variation groups according to the taxonomic relationship between the species (Figs S1–S5, Supporting information)—a finding consistent with other studies of TLR variation across species (Roach *et al*. 2005; Nakajima *et al*. 2008; Alcaide & Edwards 2011). We found no evidence of gene conversion and recombination among the TLR loci. Thus, contrary to other gene families, such as the MHC, where gene conversion and trans‐species evolution are frequent (Ohta 1995; Klein *et al*. 1998; Spurgin *et al*. 2011), vertebrate TLRs seem to evolve independently and (notwithstanding rare whole gene duplication events) by point mutation (Roach *et al*. 2005). Our results confirm that TLR loci can provide simpler and more tractable immunogenetic loci—compared to the MHC—with which to explore the forces shaping important functional diversity within and among populations. In our data the sequences from the two pipit species clustered together within the tree and share two alleles (one allele in TLR1LA and one in TLR3, Figs S1 and S3, Supporting information). However much lower interspecific TLR compared to mtDNA divergence is expected when we consider (i) the recent separation time between the two species, and (ii) the larger effective population size of nuclear compared to mitochondrial DNA. The retained ancestral polymorphism between Berthelot's and tawny pipits is therefore most likely due to incomplete lineage sorting.

There is now evidence from within and across multiple species that overall TLR sequences are characterized by stabilizing selection, but with specific TLR codons showing evidence of positive selection (Alcaide & Edwards 2011; Areal *et al*. 2011; Fornůsková *et al*. 2013; Grueber *et al*. 2014; Quéméré *et al*. 2015). In line with this, we found evidence for purifying selection at all the TLR loci amplified, but also found that specific codons were under positive selection (Table S5, Supporting information). Of particular interest in our study is the large number of selected sites detected at TLR3 using the MEME method, which models lineage‐specific selection (Murrell *et al*. 2012). In fact, the haplotype network for TLR3 (Fig. S8, Supporting information) suggests that nonsynonymous variants have been generated from a central widespread haplotype that arrived in the initial colonization of the islands, and have been selected for and maintained in the Canary Islands (four amino acid variants) and Madeira (one amino acid variant). Thus it may be this gene has undergone strong, positive selection at some point in the evolutionary history of Berthelot's and tawny pipits—however, data from a range of closely related species are required to confirm this. In general, while the d*N*/d*S* values in this study should be interpreted with caution due to the recent separation time of our species (see Kryazhimskiy & Plotkin 2008), that TLR genes are functionally important is beyond doubt.

Much less clear is how selection and drift shape patterns of TLR variation across ecological timescales in natural populations. Some recent studies provide evidence suggesting that drift is the main force shaping diversity at TLR loci in recently bottlenecked or expanding populations (Grueber *et al*. 2013; Hartmann *et al*. 2014; Quéméré *et al*. 2015). Our study indicates that even though balancing selection has played an important role in maintaining variants at some TLR loci over long‐term time frames, genetic drift can be the main driver of TLR allele frequency variation over shorter timescales encompassing the recent evolutionary history of species. The tests of selection we implemented can detect signatures of evolutionary forces that may have acted long ago. Thus the finding of codons under positive selection at TLR3 may be the result of selection on this locus previous to the recent bottlenecks that Berthelot's pipit populations went through. If signatures of selection are strong enough they will not be erased by the bottleneck events (Oliver & Piertney 2012). The lower TLR diversity in Berthelot's compared to tawny pipits is consistent with a founder effect during the initial colonization of the islands. Indeed, levels of TLR variation in Berthelot's pipit (5–10 alleles per locus) are comparable to those reported for recently bottlenecked species (Grueber *et al*. 2012), whereas TLR variation in tawny pipits (7–18 alleles per locus) is more comparable to those in widespread species (Alcaide & Edwards 2011; Quéméré *et al*. 2015). The secondary colonization of Madeira and Selvagens by Berthelot's pipits from the Canary Islands (*c*. 8500 years ago) also involved bottlenecks (Spurgin *et al*. 2014), and the lower levels of TLR variation on these archipelagos is consistent with this. Moreover, the levels of pairwise differentiation at TLR loci among Berthelot's pipit populations are extremely strongly correlated with those found at microsatellites (Fig. 3A), and are consistent with the pattern of colonization, bottlenecks and subsequent isolation in this species (Illera *et al*. 2007; Spurgin *et al*. 2014). These results indicate that the TLR variation observed across populations has largely been shaped by founder effects and that these patterns have persisted over relatively long timeframes, at least from an ecological perspective (1000s of years).

TLR genes offer an interesting comparison to MHC genes, which have been the most widely used candidate genes for studying adaptation in wild populations (Spurgin & Richardson 2010). Selection can have profound effects on MHC allele frequency variation within and across populations, even through strong population bottlenecks (e.g. Oliver & Piertney 2012). However, in other instances demographic processes can also play an important role in shaping MHC variation within and among small or bottlenecked populations (Bollmer *et al*. 2011; Girard & Angers 2011; Oliver & Piertney 2012; Sutton *et al*. 2013). To our knowledge, there is not yet any evidence of selection producing pronounced effects on TLR allele frequencies within or among natural populations, perhaps suggesting selection at TLRs is generally weaker than at MHC genes. This may be in part due to structural differences between the two gene families. In Berthelot's pipits, there are low levels of within‐island MHC variation, but functional variants have been rapidly regenerated as a result of gene conversion and selection (Spurgin *et al*. 2011). There is no evidence from this study or any other that we are aware of that gene conversion generates TLR variation. Consequently, bottlenecked populations are not able to regenerate TLR variation anywhere near the rates that can be achieved for MHC genes, reducing the efficacy with which selection can act. However, more studies of species undergoing known bouts of pathogen‐mediated selection are required to confirm this.

In conclusion, founder effects appear to have been the main determinant of patterns of TLR variation in the Berthelot's pipit. Thus even at functional loci where allelic diversity is known to be important in combating pathogens (and hence where balancing selection may be expected), drift can be the predominant force shaping variation within and among populations. Our results do not discount the importance of selection or variation at functional loci such as TLRs but rather highlight that drift can have pronounced effects on allelic variation, which need to be accurately assessed and controlled for if we are to be able to determine where and when selection has been important in wild populations.

D.S.R., L.G.S. and C.G.Q. designed the study. L.G.S., J.C.I. and D.S.R. collected samples. C.G.Q. and J.C.I. did the laboratory work. C.G.Q. and L.G.S. did the analysis. C.G.Q., D.S.R. and L.G.S. wrote the manuscript. All authors agreed on the final manuscript.

## Data accessibility

TLR and COI sequences are available from GenBank (Accession nos. KJ414322–KJ414417, KT931977–KT931992). The following data have been deposited in the DRYAD data repository doi:10.5061/dryad.9432p: Individual TLR sequences for each locus, microsatellite genotypes, PHASE results, pairwise TLR FSTs, pairwise microsatellite FSTs, r code to calculate pairwise bottleneck distances, tree files underlying each TLR phylogeny and alignment of COI sequences from *Anthus berthelotii* and *Anthus campestris*.

## Supporting information


**Figs S1–S5** Maximum likelihood phylogenetic trees of haplotypes at five TLR loci in five bird species: *Came* = house finch, *Carpodacus mexicanus*;* Peau* = New Zealand robin, *Petroica australis rakiura*;* Fana* = Lesser kestrel, *Falco naumanni*;* Anbe* = Berthelot's pipit, *Anthus berthelotii,* and *Anca* = tawny pipit, *Anthus campestris*. Figure S1 = TLR1LA, S2 = TLR1LB, S3 = TLR3, S4 = TLR4, S5 = TLR21. Node values represent bootstrap support. Subtrees for *Peau, Anca* and *Fana* were collapsed. Height of the collapsed subtree is proportional to the number of haplotypes in the subtree.

**Figs S6–S10** Networks of TLR haplotypes found in populations of Berthelot's pipits (Yellow: Canary Islands, Blue: Selvagens, Green: Madeira) and in tawny pipits (white circles). Each circle represents one haplotype. Connections between circles denote the number of nucleotide substitutions needed to change from one haplotype to another. Nonsynonymous substitutions are marked in red. Haplotype number is denoted beside each circle and size of the circle is proportional to the abundance of the haplotype in Berthelot's pipits. Circles representing tawny pipit haplotypes are drawn at a standard size, and are for comparison of relationships with Berthelot's pipit haplotypes only.

**Appendix S1** Methodology for obtaining sequences of cytochrome oxidase subunit I (COI).
**Table S1** Number of individuals screened per island at each TLR locus.
**Table S2** Variation at the exons encoding the extracellular domains of five toll‐like receptor genes in Berthelot's pipit (*Anthus berthelotii*).
**Table S3** Haplotypes identified at the five TLR loci in Berthelot's pipit, A*nthus berthelotii* and tawny pipit, *A. campestris*, populations.
**Table S4** Mean ratio of nonsynonymous to synonymous substitutions (ω, *P* values for hypothesis of purifying selection, d*N* < d*S*, in brackets) across identified alleles of the five TLR loci in 1) Berthelot's pipit (Aber) and 2) both Berthelot's and tawny pipits (Aber + Acam).
**Table S5** Codons in TLR1LA, TLR3 and TLR4 identified as being under positive selection across Berthelot's and tawny pipits, using two different methods: FUBAR and MEME.
