# Supplementary figures and images for: Drift, not selection, shapes toll‐like receptor variation among oceanic island populations
Source: Mol Ecol. 2015 Nov 24;24(23):5852–63. doi: 10.1111/mec.13437 (PMC4737395; doi:10.1111/mec.13437)

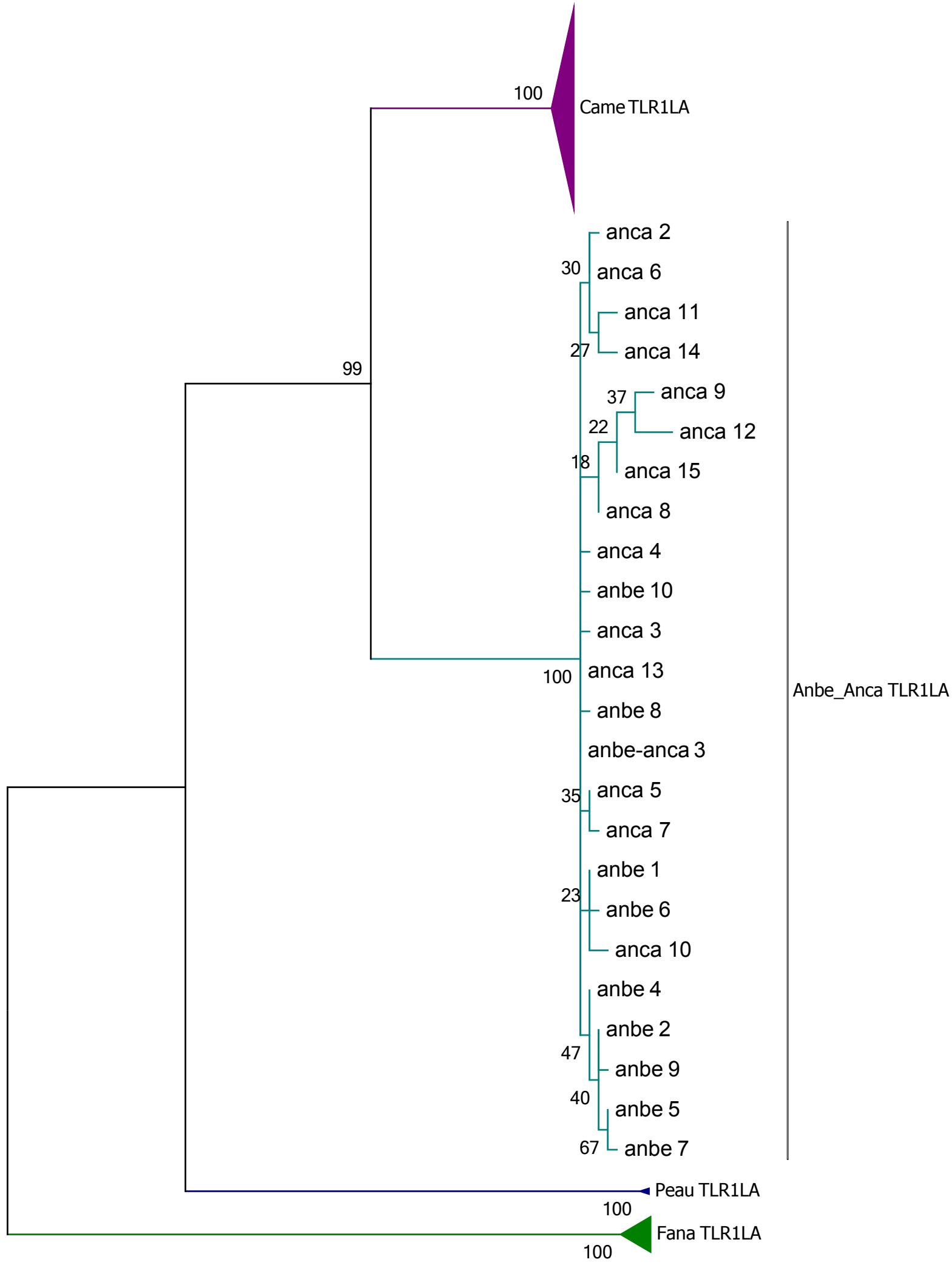

Anbe\_Ancal TLR1LA

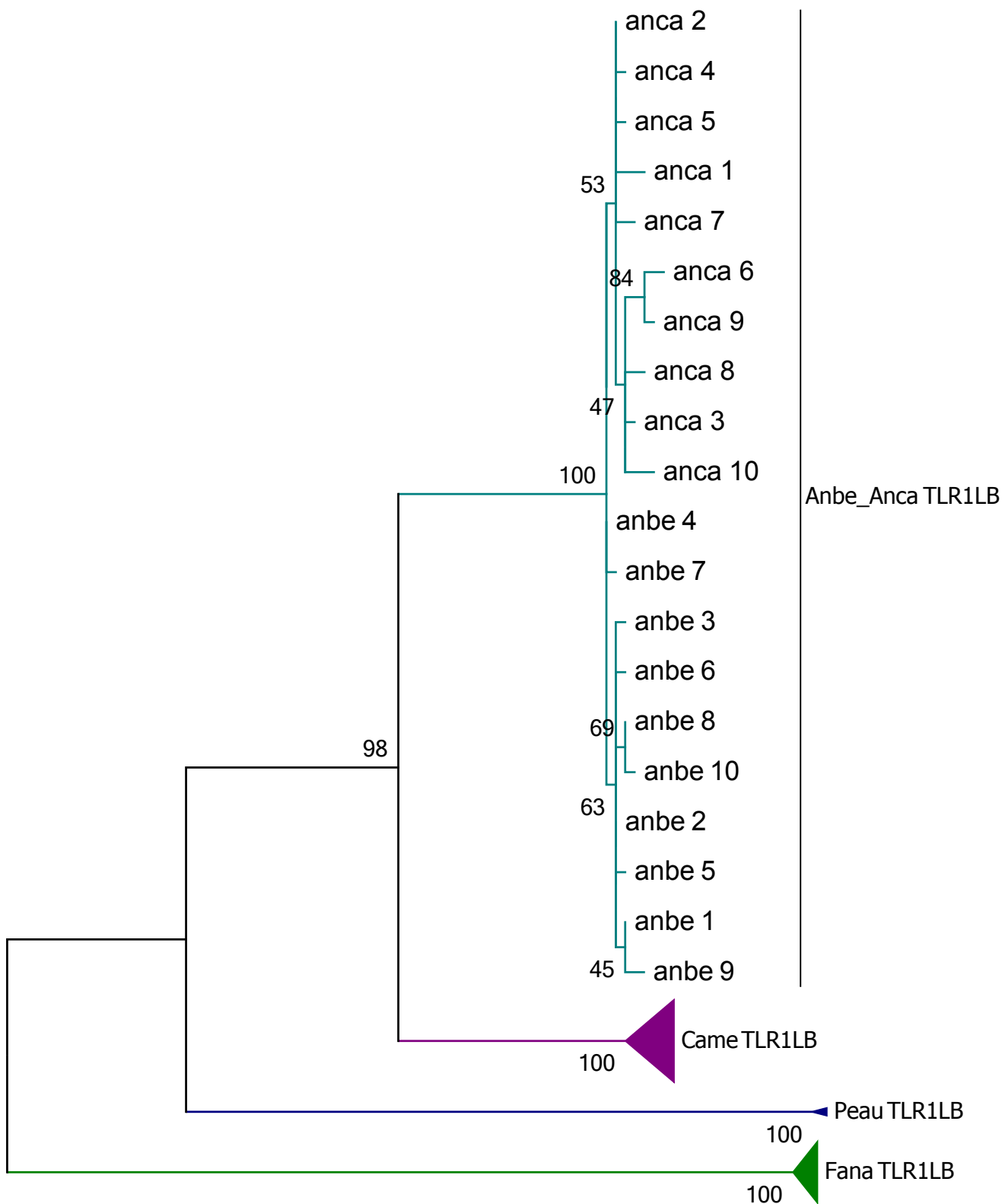

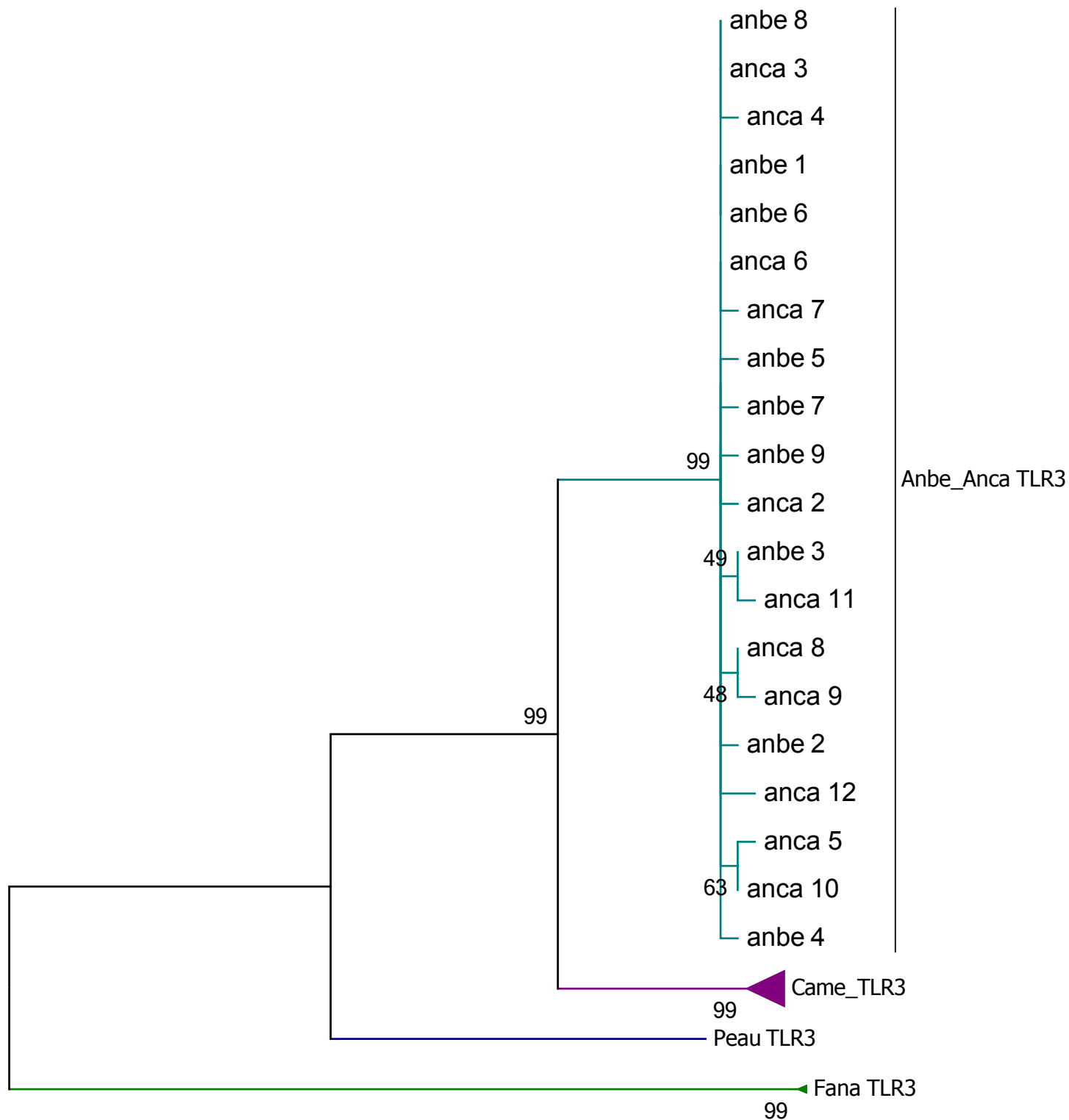

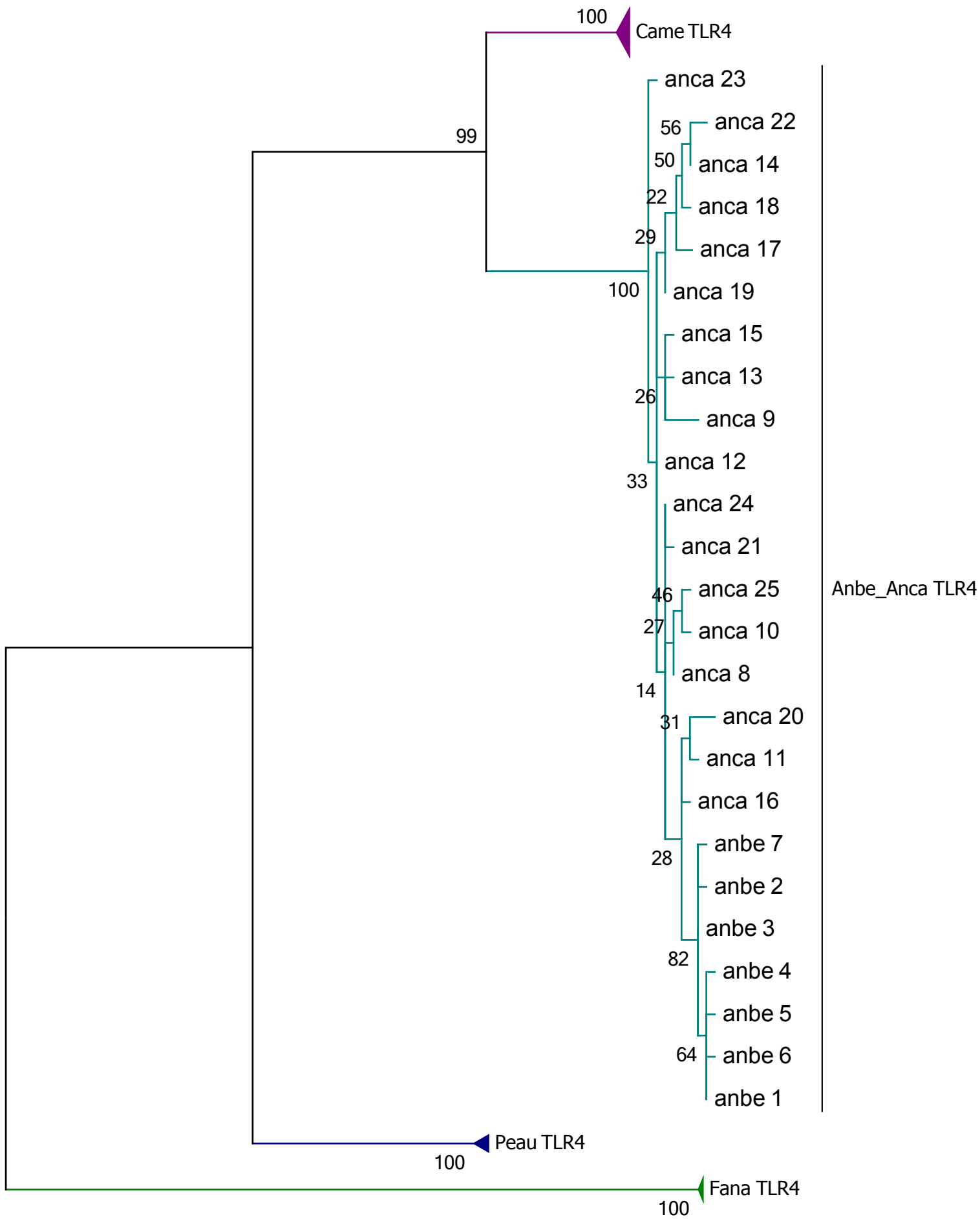

0.02

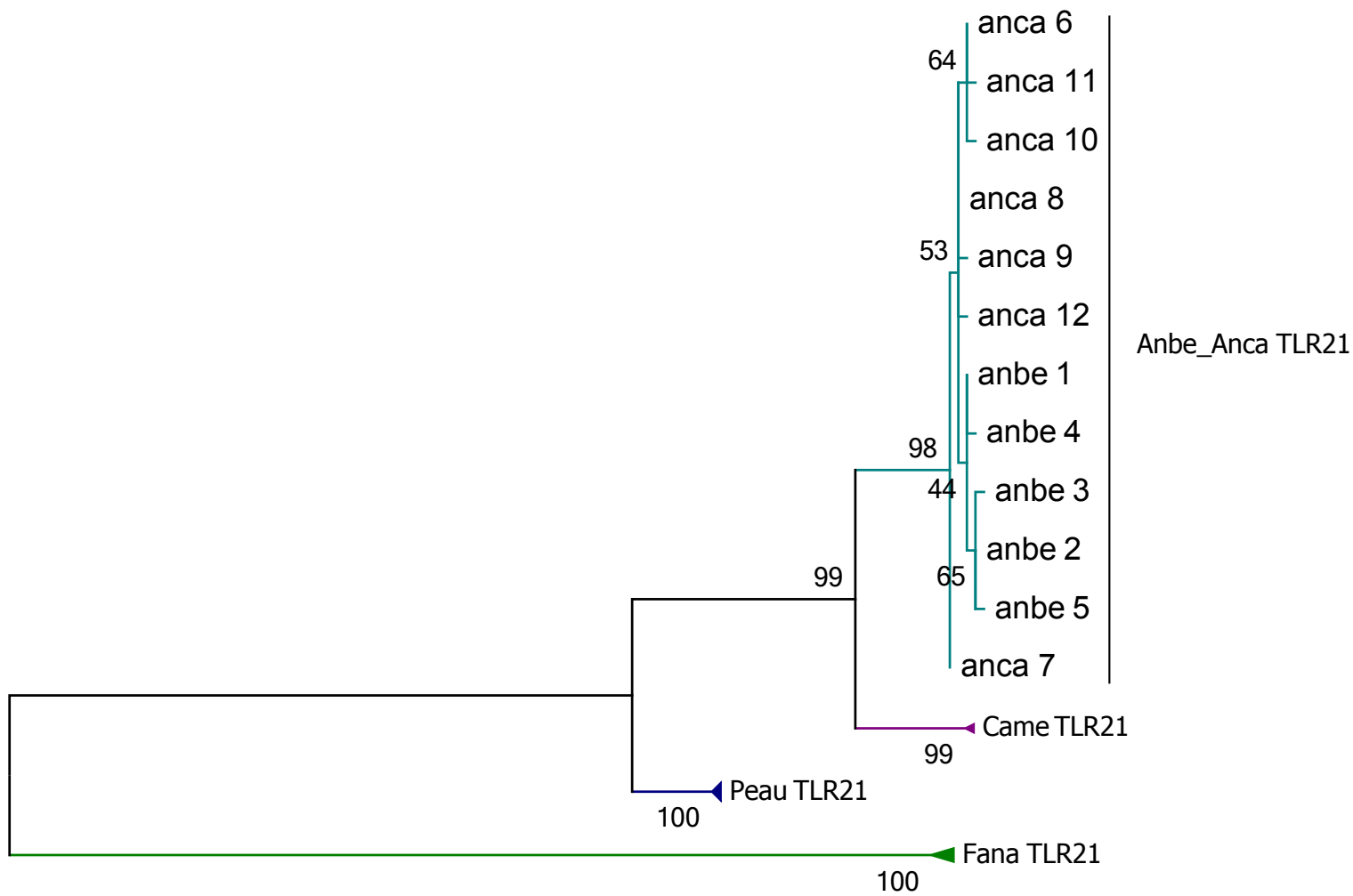

Supplement: Supplementary file 1 — Figs S1–S5 Maximum likelihood phylogenetic trees of haplotypes at five TLR loci in five bird species: Came = house finch, Carpodacus mexicanus; Peau = New Zealand robin, Petroica australis rakiura; Fana = Lesser kestrel, Falco naumanni; Anbe = Berthelot's pipit, Anthus berthelotii, and Anca = tawny pipit, Anthus campestris. Figure S1 = TLR1LA, S2 = TLR1LB, S3 = TLR3, S4 = TLR4, S5 = TLR21. Node values represent bootstrap support. Subtrees for Peau, Anca and Fana were collapsed. Height of the collapsed subtree is proportional to the number of haplotypes in the subtree. [file MEC-24-5852-s001.pdf]
