# Supplementary material for: Drift, not selection, shapes toll‐like receptor variation among oceanic island populations
Source: Mol Ecol. 2015 Nov 24;24(23):5852–63. doi: 10.1111/mec.13437 (PMC4737395; doi:10.1111/mec.13437)
Supplement: Supplementary file 2 — Figs S6–S10 Networks of TLR haplotypes found in populations of Berthelot's pipits (Yellow: Canary Islands, Blue: Selvagens, Green: Madeira) and in tawny pipits (white circles). Each circle represents one haplotype. Connections between circles denote the number of nucleotide substitutions needed to change from one haplotype to another. Nonsynonymous substitutions are marked in red. Haplotype number is denoted beside each circle and size of the circle is proportional to the abundance of the haplotype in Berthelot's pipits. Circles representing tawny pipit haplotypes are drawn at a standard size, and are for comparison of relationships with Berthelot's pipit haplotypes only. [file MEC-24-5852-s002.pdf]

TLR1LA

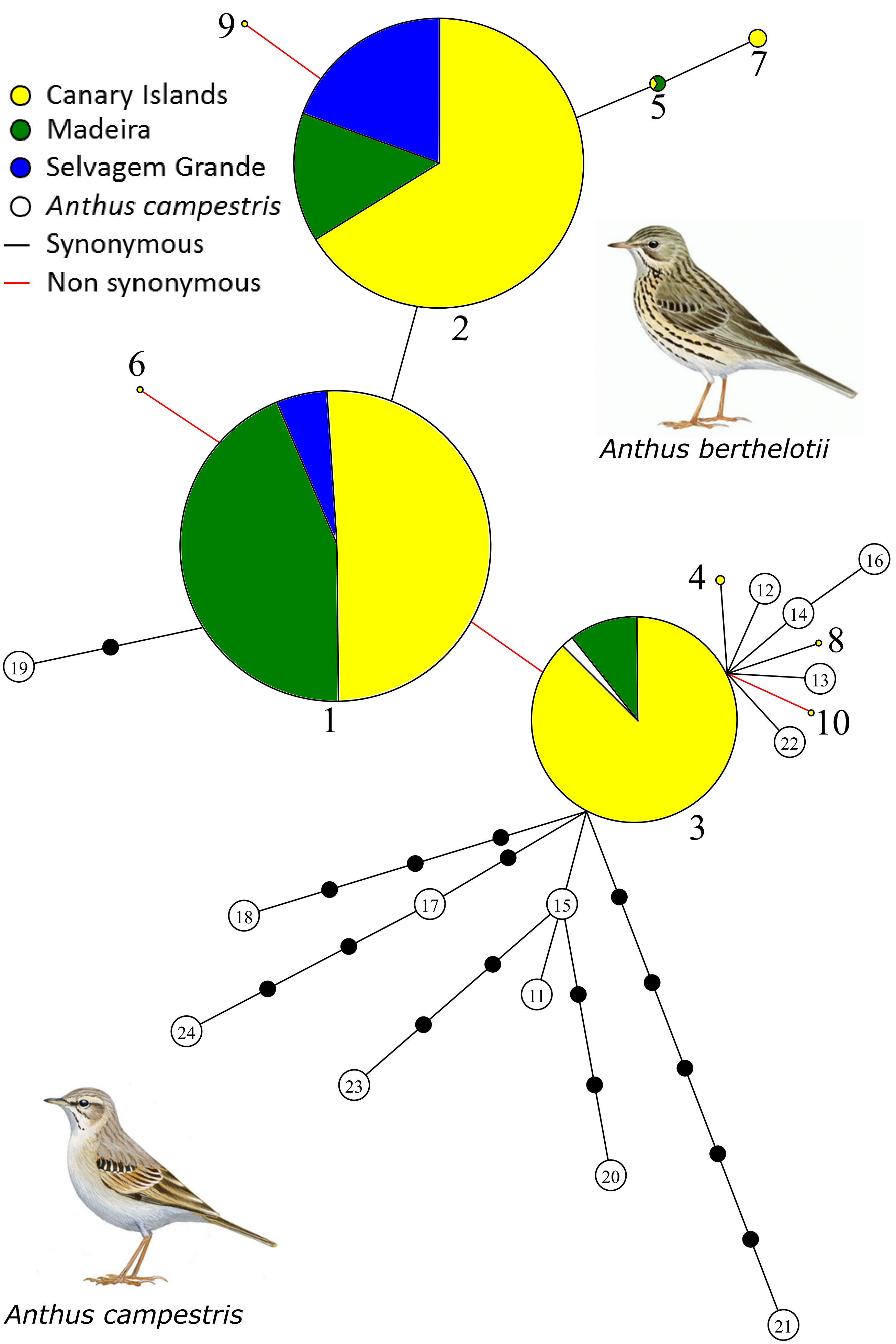

Canary Islands
Madeira
Selvagem Grande
Anthus campestris
Synonymous
Non synonymous
1
2
3
4
5
6
7
8
9
10
11
12
13
14
15
16
17
18
19
20
21
22
23
24
Anthus berthelotii
Anthus campestris

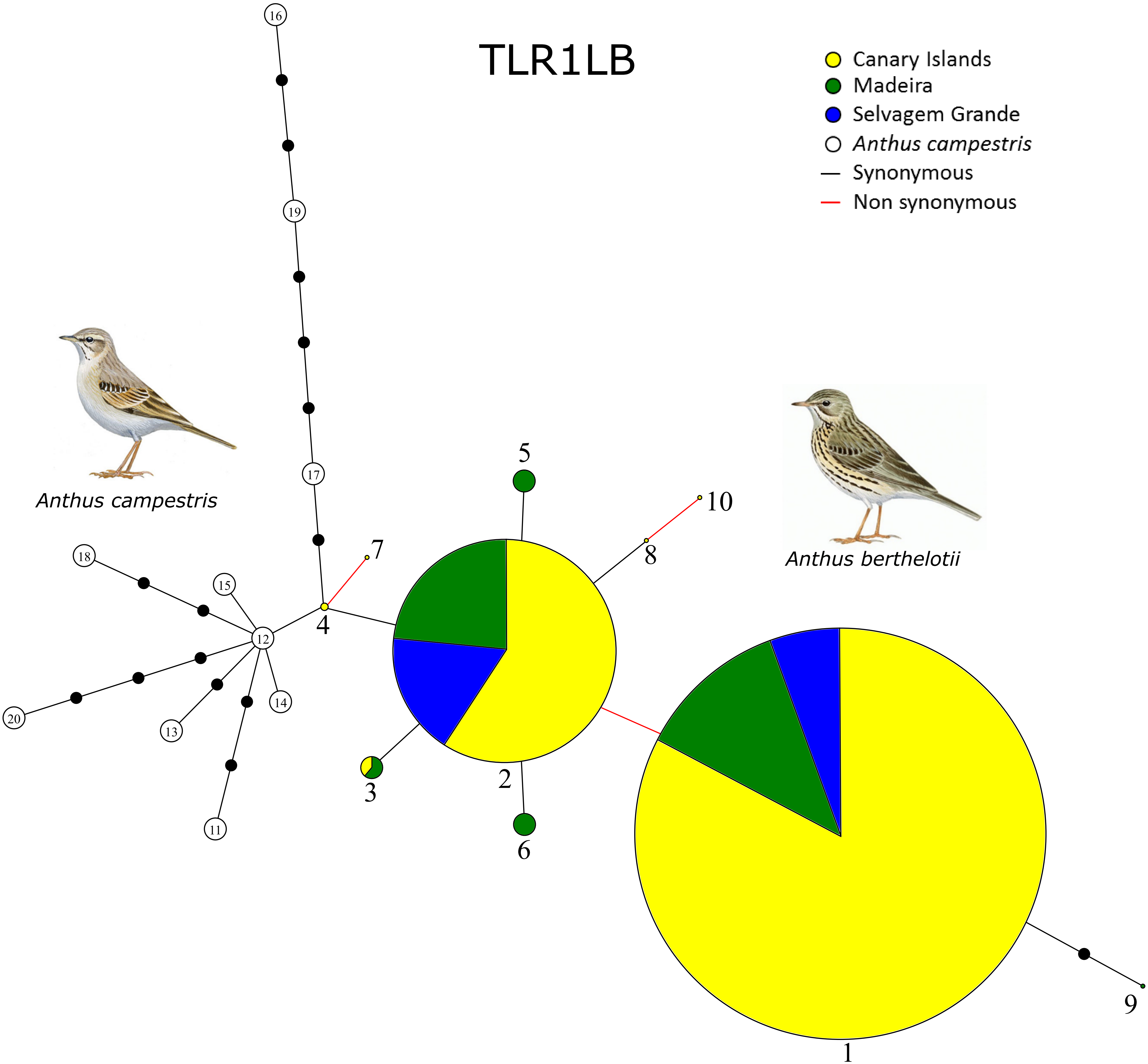
TLR1LB
Canary Islands
Madeira
Selvagem Grande
Anthus campestris
Synonymous
Non synonymous
Anthus campestris
Anthus berthelotii
1
2
3
4
5
6
7
8
9
10
11
12
13
14
15
16
17
18
19
20

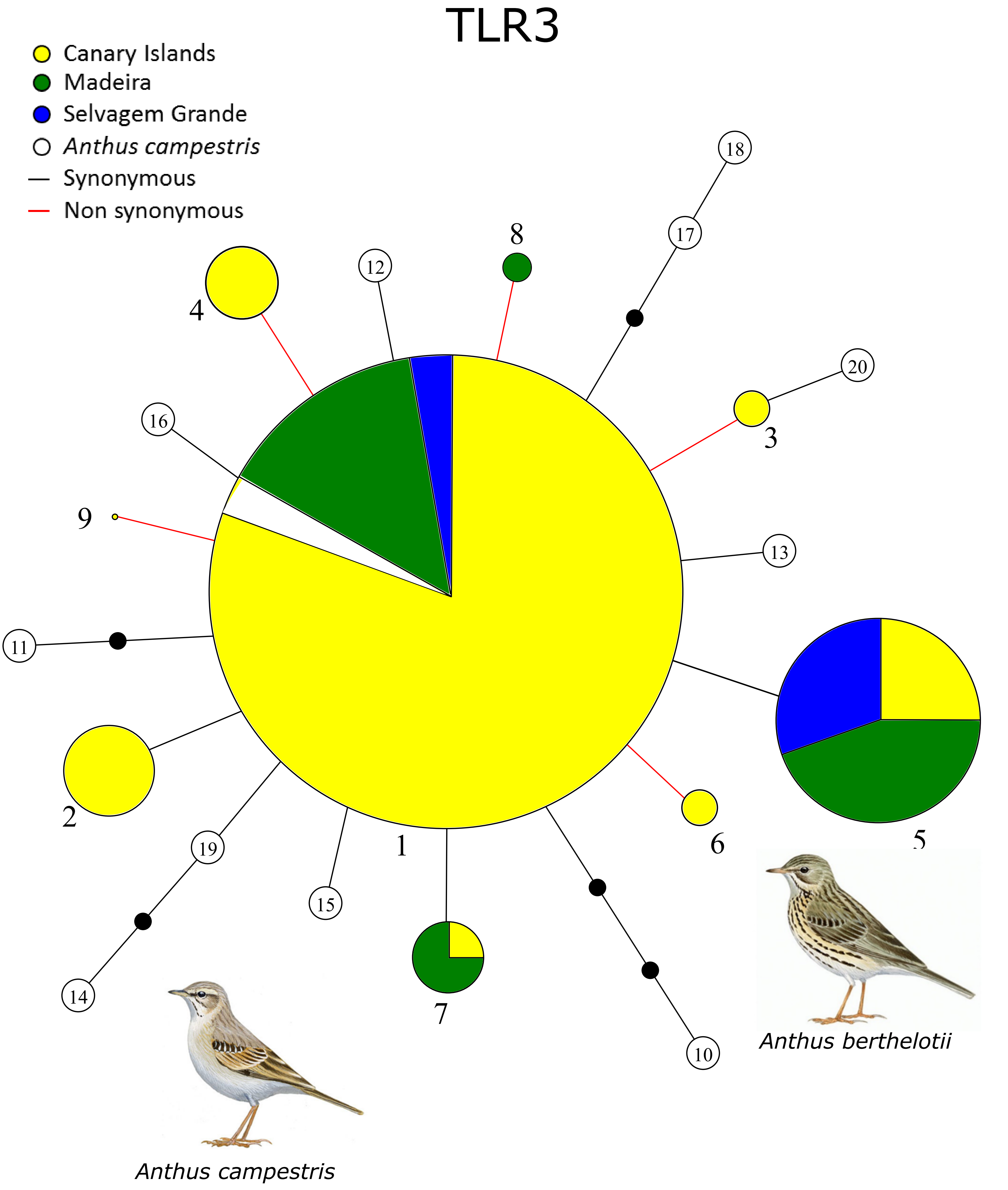

TLR3
Canary Islands
Madeira
Selvagem Grande
Anthus campestris
Synonymous
Non synonymous
1
2
3
4
5
6
7
8
9
10
11
12
13
14
15
16
17
18
19
20
Anthus berthelotii
Anthus campestris

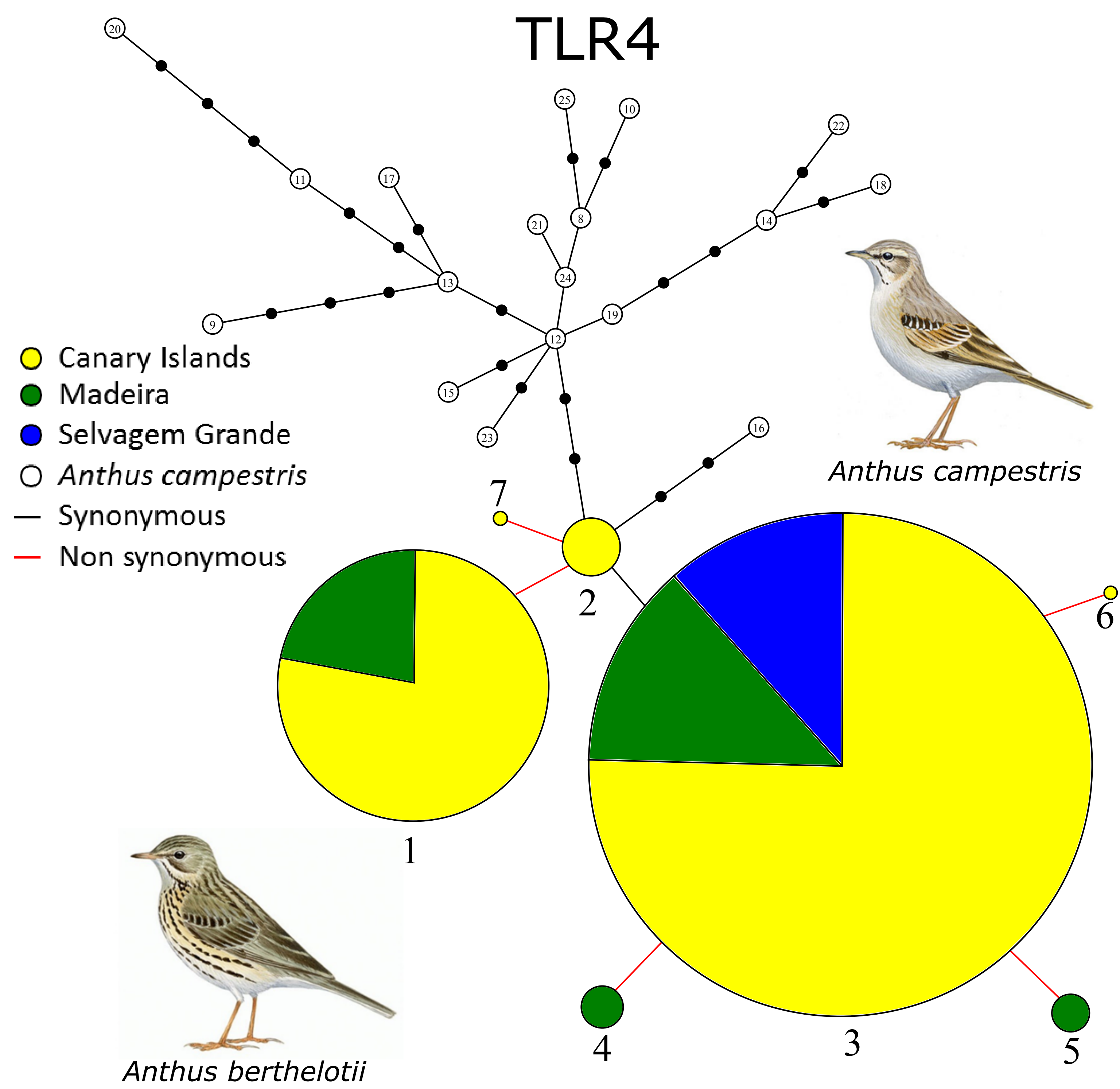
TLR4
Canary Islands
Madeira
Selvagem Grande
Anthus campestris
Synonymous
Non synonymous
Anthus campestris
Anthus berthelotii
1
2
3
4
5
6
7
8
9
10
11
12
13
14
15
16
17
18
19
20
21
22
23
24
25

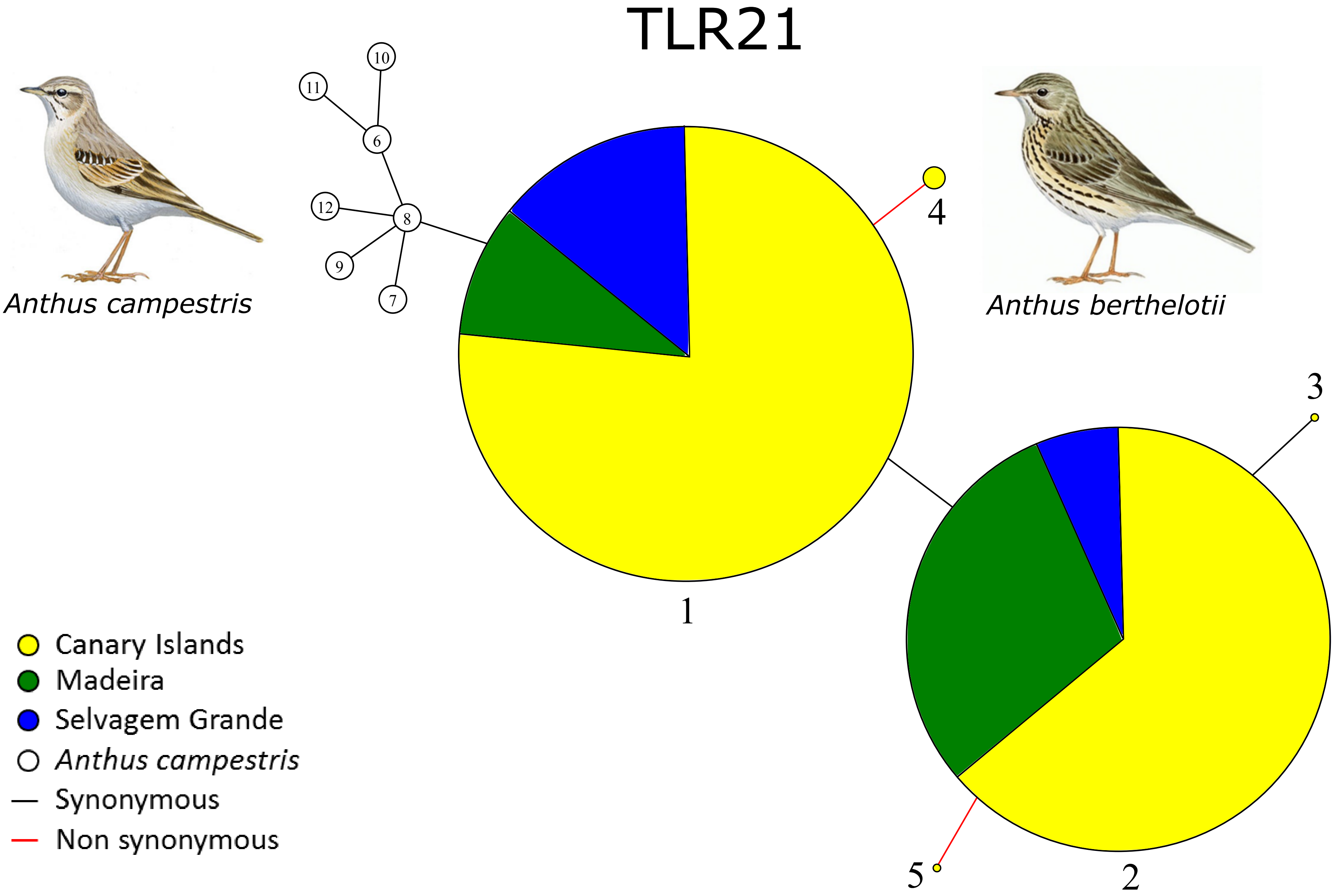

TLR21
Anthus campestris
Anthus berthelotii
10
11
6
12
8
9
7
4
3
1
2
5
Canary Islands
Madeira
Selvagem Grande
Anthus campestris
Synonymous
Non synonymous
