# Supplementary material for: Drift, not selection, shapes toll‐like receptor variation among oceanic island populations
Source: Mol Ecol. 2015 Nov 24;24(23):5852–63. doi: 10.1111/mec.13437 (PMC4737395; doi:10.1111/mec.13437)
Supplement: Supplementary file 3 — Appendix S1 Methodology for obtaining sequences of cytochrome oxidase subunit I (COI). Table S1 Number of individuals screened per island at each TLR locus. Table S2 Variation at the exons encoding the extracellular domains of five toll‐like receptor genes in Berthelot's pipit (Anthus berthelotii). Table S3 Haplotypes identified at the five TLR loci in Berthelot's pipit, Anthus berthelotii and tawny pipit, A. campestris, populations. Table S4 Mean ratio of nonsynonymous to synonymous substitutions (ω, P values for hypothesis of purifying selection, dN < dS, in brackets) across identified alleles of the five TLR loci in 1) Berthelot's pipit (Aber) and 2) both Berthelot's and tawny pipits (Aber + Acam). Table S5 Codons in TLR1LA, TLR3 and TLR4 identified as being under positive selection across Berthelot's and tawny pipits, using two different methods: FUBAR and MEME. [file MEC-24-5852-s003.docx]

The following supplementary material is available for this article online.

Supplementary text

*Methodology for obtaining sequences of cytochrome oxidase subunit I (COI)*

A 655 bp fragment of the COI gene was amplified in nine tawny pipits (four from northern Africa, including two confirmed resident birds from Morocco, and five from the Iberian Peninsula) and sixteen Berthelot’s pipits (ten from the Canary Islands, three from Selvagens and three from Madeira) using primers PasserF1 and PasserR1 ([Lohman *et al.* 2009](#_ENREF_46)) following thermal conditions described in Lohman *et al.* (2009). Amplification was carried out in 10 µl reactions using a Tetrad thermocycler (MJ Research), including TopTaq polymerase master mix (Qiagen), 0.5 µM of each primer and ca. 30 ng of DNA. Amplified fragments were visualized in 2% agarose gels stained with ethidium bromide and purified using a mixture of recombinant alkaline phosphatase and exonuclease I, incubating at 37°C for 30 mins, followed by enzyme inactivation at 95°C for 5 mins. Amplified fragments were sequenced using the BigDye terminator kit (Applied Biosystems) using the following thermal profile: 96°C for 2 mins, followed by 25 cycles of 96°C for 10 s, 50°C for 5 s and 60°C for 4 mins. Products were visualized in an ABI genetic analyzer (Applied Biosystems). Sequences were aligned and edited in BioEdit 7.0.9.0 ([Hall 1999](#_ENREF_32)). For divergence time estimates we included a further five sequences of tawny pipit COI published in GenBank (Accession numbers GQ481330-34). The software BEAST v 1.8.0 ([Drummond & Rambaut 2007](#_ENREF_16)) was used to estimate divergence times between COI sequences of Berthelot’s and tawny pipits using constant size population priors, the Hasegawa-Kishino-Yano (HKY) nucleotide substitution model and a strict clock with a divergence rate of 2.1% per million years ([Weir & Schluter 2008](#_ENREF_86)). The software was run for 10 million generations with a burn in of 1 million generations. The software Tracer v 1.5 ([Rambaut & Drummond 2009](#_ENREF_67)) was then used to assess convergence of the chains and to obtain the mean and 95% intervals of highest posterior density (HPD) estimates of divergence time.

Table S1. Number of individuals screened per island at each TLR locus.

| Archipelago | Island | TLR1LA | TLR1LB | TLR3 | TLR4 | TLR21 |
| --- | --- | --- | --- | --- | --- | --- |
| Canary Islands | El Hierro | 10 | 10 | 29 | 30 | 10 |
| Canary Islands | Fuerte Ventura | 10 | 10 | 30 | 30 | 10 |
| Canary Islands | Gran Canaria | 10 | 10 | 30 | 30 | 10 |
| Canary Islands | La Gomera | 14 | 16 | 28 | 27 | 10 |
| Canary Islands | Graciosa | 10 | 10 | 30 | 30 | 10 |
| Canary Islands | La Palma | 10 | 10 | 30 | 29 | 10 |
| Canary Islands | Lanzarote | 10 | 6 | 31 | 32 | 10 |
| Canary Islands | Teide | 13 | 12 | 28 | 27 | 13 |
| Canary Islands | Tenerife | 13 | 15 | 30 | 30 | 15 |
| Madeira | Deserta Grande | 10 | 10 | 28 | 30 | 10 |
| Madeira | Madeira | 11 | 11 | 22 | 23 | 10 |
| Madeira | Porto Santo | 11 | 11 | 30 | 30 | 10 |
| Selvagens | Selvagem Grande | 14 | 14 | 25 | 23 | 17 |

Table S2. Variation at the exons encoding the extracellular domains of five toll-like receptor genes in Berthelot’s pipit (*Anthus berthelotii*).

| Locus (exon) | N^1^ | Fragment length (bp) | SNPs^2^ | Amino acid | Syn:nsyn^3^ | H^4^ | Amino acid  variants | π x10^-4^ (SD)^5^ |
| --- | --- | --- | --- | --- | --- | --- | --- | --- |
| TLR1LA (2) | 134 | 1011 | 70: C/T | Leu | 4:4 | 10 | 5 | 24.2 (3.3) |
|  |  |  | 243: C/T | Ser/Phe |  |  |  |  |
|  |  |  | 284:C/T | Arg/Cys |  |  |  |  |
|  |  |  | 322:C/T | Tyr |  |  |  |  |
|  |  |  | 657:C/T | Phe/Ser |  |  |  |  |
|  |  |  | 871:C/T | Leu |  |  |  |  |
|  |  |  | 925:C/T | Ala |  |  |  |  |
|  |  |  | 986:C/T | Ser/Pro |  |  |  |  |
| TLR1LB (1) | 141 | 975 | 327:C/T | Cys | 6:3 | 10 | 5 |  |
|  |  |  | 518:C/T | Phe/Ser |  |  |  |  |
|  |  |  | 675:A/G | Pro |  |  |  |  |
|  |  |  | 732:C/T | Leu |  |  |  |  |
|  |  |  | 798:A/G | Pro |  |  |  |  |
|  |  |  | 833:A/G | Arg/His |  |  |  |  |
|  |  |  | 873:A/G | Pro |  |  |  |  |
|  |  |  | 915:A/G | Thr |  |  |  |  |
|  |  |  | 944:A/T | Leu/Gln |  |  |  |  |
| TLR3 (4) | 371 | 1041 | 38:C/T | Ser/Phe | 3:5 | 9 | 6 | 17.1 (1.8) |
|  |  |  | 88:A/G | Ile/Val |  |  |  |  |
|  |  |  | 197:C/T | Ser/Leu |  |  |  |  |
|  |  |  | 199:C/T | Arg/Cys |  |  |  |  |
|  |  |  | 573:C/T | Gly |  |  |  |  |
|  |  |  | 696:A/G | Val |  |  |  |  |
|  |  |  | 748:C/T | Leu |  |  |  |  |
|  |  |  | 814:C/G | Asp/His |  |  |  |  |
| TLR4 (3) | 372 | 660 | 215:A/G | Asp/Gly | 1:4 | 7 | 6 | 29.6 (4.5) |
|  |  |  | 280:A/G | Lys/Glu |  |  |  |  |
|  |  |  | 300:C/T | Pro |  |  |  |  |
|  |  |  | 302:A/C/T | Lys/Thr/Met |  |  |  |  |
|  |  |  | 320:A/G | Glu/Arg |  |  |  |  |
| TLR21 (1) | 144 | 622 | 247:C/T | Asp | 2:2 | 5 | 3 | 28.9 (6.2) |
|  |  |  | 554:C/T | Leu/Cys |  |  |  |  |
|  |  |  | 579:C/G | Gly/Ala |  |  |  |  |
|  |  |  | 607:C/T | Phe |  |  |  |  |
| Mean |  | 861.8 |  |  | 6.4:3.6 | 8.4 | 5 |  |

^1^Number of individuals genotyped

^2^Single nucleotide polymorphisms. Number indicates nucleotide position in the sequence.

^3^Number of synonymous to number of nonsynonymous mutations

^4^Number of haplotypes

^5^Nucleotide diversity (Standard deviation)

Table S3. Haplotypes identified at the five TLR loci in Berthelot’s pipit, A*nthus berthelotii* and tawny pipit, *A. campestris*, populations. CI = Canary Islands, M = Madeira, S = Selvagem Grande, IP = Iberian Peninsula, Af = north Africa. Only variable sites are shown in the sequence.

| *A. berthelotii*  allele | Sequence | Amino acid variant | N^1^ | Frequency^2^ | Frequency  In CI | Frequency in M | Frequency in S | GenBank  Accession # |
| --- | --- | --- | --- | --- | --- | --- | --- | --- |
| TLR1LA_1 | CCCCTCCT | TLR1LA-1 | 106 | 0.355 | 0.285 | 0.594 | 0.357 | KJ414322 |
| TLR1LA_2 | TCCCTCCT | TLR1LA-1 | 99 | 0.332 | 0.315 | 0.219 | 0.643 | KJ414323 |
| TLR1LA_3 | CCCCCCCT | TLR1LA-2 | 70 | 0.235 | 0.310 | 0.109 | 0.000 | KJ414324 |
| TLR1LA_4 | TCCCCCCT | TLR1LA-2 | 3 | 0.010 | 0.015 | 0.000 | 0.000 | KJ414325 |
| TLR1LA_5 | TCCCTCTT | TLR1LA-1 | 7 | 0.023 | 0.010 | 0.078 | 0.000 | KJ414326 |
| TLR1LA_6 | CCTCTCCT | TLR1LA-3 | 2 | 0.006 | 0.010 | 0.000 | 0.000 | KJ414327 |
| TLR1LA_7 | TCCCTTTT | TLR1LA-1 | 6 | 0.020 | 0.030 | 0.000 | 0.000 | KJ414328 |
| TLR1LA_8 | CCCTCCCT | TLR1LA-2 | 1 | 0.003 | 0.005 | 0.000 | 0.000 | KJ414329 |
| TLR1LA_9 | TCCCTCCC | TLR1LA-4 | 2 | 0.006 | 0.010 | 0.000 | 0.000 | KT931986 |
| TLR1LA_10 | CTCCCCCT | TLR1LA-5 | 2 | 0.006 | 0.010 | 0.000 | 0.000 | KT931987 |
| TLR1LB_1 | CTGCGGGGT | TLR1LB-1 | 153 | 0.527 | 0.636 | 0.281 | 0.321 | KJ414330 |
| TLR1LB_2 | CCGCGGGGT | TLR1LB-2 | 104 | 0.358 | 0.308 | 0.375 | 0.679 | KJ414331 |
| TLR1LB_3 | CCGCAGGGT | TLR1LB-2 | 10 | 0.034 | 0.020 | 0.094 | 0.000 | KJ414332 |
| TLR1LB_4 | TCGCGGGGT | TLR1LB-2 | 3 | 0.010 | 0.015 | 0.000 | 0.000 | KJ414333 |
| TLR1LB_5 | CCACGGGGT | TLR1LB-2 | 10 | 0.034 | 0.000 | 0.156 | 0.000 | KJ414334 |
| TLR1LB_6 | CCGCGGAGT | TLR1LB-2 | 4 | 0.014 | 0.000 | 0.063 | 0.000 | KJ414335 |
| TLR1LB_7 | TCGCGAGGT | TLR1LB-3 | 1 | 0.003 | 0.005 | 0.000 | 0.000 | KJ414336 |
| TLR1LB_8 | CCGCGGGAT | TLR1LB-2 | 2 | 0.007 | 0.010 | 0.000 | 0.000 | KJ414337 |
| TLR1LB_9 | CTATGGGGT | TLR1LB-4 | 2 | 0.007 | 0.000 | 0.031 | 0.000 | KT931988 |
| TLR1LB_10 | CCGCGGGAA | TLR1LB-5 | 1 | 0.003 | 0.005 | 0.000 | 0.000 | KT931989 |
| TLR3_1 | CACCTACG | TLR3-1 | 436 | 0.588 | 0.671 | 0.413 | 0.260 | KJ414338 |
| TLR3_2 | CACCTATG | TLR3-1 | 51 | 0.069 | 0.096 | 0.000 | 0.000 | KJ414339 |
| TLR3_3 | CATCTACG | TLR3-2 | 21 | 0.028 | 0.039 | 0.000 | 0.000 | KJ414340 |
| TLR3_4 | CACCTACC | TLR3-3 | 41 | 0.055 | 0.077 | 0.000 | 0.000 | KJ414341 |
| TLR3_5 | CACCCACG | TLR3-1 | 115 | 0.155 | 0.055 | 0.306 | 0.740 | KJ414342 |
| TLR3_6 | CGCCTACG | TLR3-4 | 20 | 0.027 | 0.038 | 0.000 | 0.000 | KJ414343 |
| TLR3_7 | CACCTGCG | TLR3-1 | 39 | 0.052 | 0.019 | 0.181 | 0.000 | KT931990 |
| TLR3_8 | TACCTACG | TLR3-5 | 16 | 0.022 | 0.000 | 0.100 | 0.000 | KT931991 |
| TLR3_9 | CACTTACG | TLR3-6 | 3 | 0.004 | 0.006 | 0.000 | 0.000 | KT931992 |
| TLR4_1 | AGCCA | TLR4-1 | 398 | 0.535 | 0.312 | 0.295 | 0.000 | KJ414344 |
| TLR4_2 | AGTAA | TLR4-2 | 215 | 0.289 | 0.086 | 0.000 | 0.000 | KJ414345 |
| TLR4_3 | AGTCA | TLR4-1 | 46 | 0.062 | 0.562 | 0.319 | 1.000 | KJ414346 |
| TLR4_4 | GGCCA | TLR4-3 | 30 | 0.040 | 0.000 | 0.205 | 0.000 | KJ414347 |
| TLR4_5 | AACCA | TLR4-4 | 34 | 0.046 | 0.000 | 0.181 | 0.000 | KJ414348 |
| TLR4_6 | AGCCG | TLR4-5 | 11 | 0.015 | 0.021 | 0.000 | 0.000 | KJ414349 |
| TLR4_7 | AGTTA | TLR4-6 | 10 | 0.013 | 0.019 | 0.000 | 0.000 | KJ414350 |
| TLR21_1 | CCGC | TLR21-1 | 145 | 0.503 | 0.521 | 0.317 | 0.735 | KJ414351 |
| TLR21_2 | CCGT | TLR21-1 | 138 | 0.479 | 0.454 | 0.683 | 0.265 | KJ414352 |
| TLR21_3 | TCGT | TLR21-1 | 1 | 0.003 | 0.005 | 0.000 | 0.000 | KJ414353 |
| TLR21_4 | CCCC | TLR21-2 | 3 | 0.010 | 0.015 | 0.000 | 0.000 | KJ414354 |
| TLR21_5 | CTGT | TLR21-3 | 1 | 0.003 | 0.005 | 0.000 | 0.000 | KJ414355 |
| Total | 41 | 25 |  |  |  |  |  |  |

| *A. campestris*  allele | **Sequence** | **Amino acid**  **variant** | **N^1^** | **Frequency^2^** | **Frequency**  **in IP** | **Frequency**  **in Af** | **Genebank**  **Accession #** |
| --- | --- | --- | --- | --- | --- | --- | --- |
| TLR1LA_1 | GAAGTAGTCCGCTCCCGACCC | TLR1LA_1 | 1 | 0.04 | 0.00 | 0.08 | KJ414356 |
| TLR1LA_3 | GAAGTAGTCCGCCCCCGACCC | TLR1LA_2 | 6 | 0.25 | 0.25 | 0.25 | KJ414357 |
| TLR1LA_9 | GAAGTAGACCGCCCCCGACCC | TLR1LA_3 | 1 | 0.04 | 0.08 | 0.00 | KJ414358 |
| TLR1LA_10 | GAAGTAGTCCGCCTCCGACCC | TLR1LA_4 | 1 | 0.04 | 0.08 | 0.00 | KJ414359 |
| TLR1LA_11 | GACGTAGTCCGCCCCCGACCC | TLR1LA_5 | 2 | 0.08 | 0.17 | 0.00 | KJ414360 |
| TLR1LA_12 | GCAGTAGTCCGCCCCCGACCC | TLR1LA_2 | 3 | 0.08 | 0.17 | 0.08 | KJ414361 |
| TLR1LA_13 | GAAGTAGTCCGCCCCCGCCCC | TLR1LA_6 | 1 | 0.04 | 0.08 | 0.00 | KJ414362 |
| TLR1LA_14 | GAAGTAGTCCGTCCCCGACCC | TLR1LA_2 | 1 | 0.04 | 0.08 | 0.00 | KJ414363 |
| TLR1LA_15 | GAAGTAGTATGCCCCCTCCTC | TLR1LA_7 | 1 | 0.04 | 0.08 | 0.00 | KJ414364 |
| TLR1LA_16 | GACGTAGTCCGCCCCCGATCC | TLR1LA_5 | 1 | 0.04 | 0.00 | 0.08 | KJ414365 |
| TLR1LA_17 | GCAATAGTCTGCCCCCGCCCC | TLR1LA_6 | 1 | 0.04 | 0.00 | 0.08 | KJ414366 |
| TLR1LA_18 | GAAGCTGTACATCCCCGCCCC | TLR1LA_8 | 1 | 0.04 | 0.00 | 0.08 | KJ414367 |
| TLR1LA_19 | AAAGTAGTCCGCCCCCGACCC | TLR1LA_2 | 2 | 0.08 | 0.00 | 0.17 | KJ414368 |
| TLR1LA_20 | GCAGTAATCCGCCCCTGCCCC | TLR1LA_6 | 1 | 0.04 | 0.00 | 0.08 | KJ414369 |
| TLR1LA_21 | GAAGTAGTATGCCCCCTCCCT | TLR1LA_9 | 1 | 0.04 | 0.00 | 0.08 | KJ414370 |
| TLR1LB_9 | GCGCAAGTGCCCTCGGAC | TLR1LB_1 | 15 | 0.63 | 0.58 | 0.67 | KJ414371 |
| TLR1LB_10 | CTGCAAGTGCCCTTGGAC | TLR1LB_2 | 1 | 0.04 | 0.08 | 0.00 | KJ414372 |
| TLR1LB_11 | GCGTAAGTGCCCTCGGCC | TLR1LB_3 | 1 | 0.04 | 0.08 | 0.00 | KJ414373 |
| TLR1LB_12 | GCGCAAGTACCCTCGGAC | TLR1LB_1 | 1 | 0.04 | 0.08 | 0.00 | KJ414374 |
| TLR1LB_13 | GCGCAAGTGCCCCCGGAC | TLR1LB_1 | 1 | 0.04 | 0.08 | 0.00 | KJ414375 |
| TLR1LB_14 | GCGCGAGTGTCTTCGGCT | TLR1LB_3 | 1 | 0.04 | 0.08 | 0.00 | KJ414376 |
| TLR1LB_15 | GCACAAGTGTCCTCGGAC | TLR1LB_4 | 1 | 0.04 | 0.00 | 0.08 | KJ414377 |
| TLR1LB_16 | GCGCACGTGCCCTCAGCC | TLR1LB_3 | 1 | 0.04 | 0.00 | 0.08 | KJ414378 |
| TLR1LB_17 | GCGCAAGTGTTTTCGGCC | TLR1LB_3 | 1 | 0.04 | 0.00 | 0.08 | KJ414379 |
| TLR1LB_18 | GCGCAAAAGCCCTCGTCC | TLR1LB_5 | 1 | 0.04 | 0.00 | 0.08 | KJ414380 |
| TLR3_2 | AGTTAGCGGCGCGCC | TLR3_1 | 12 | 0.50 | 0.58 | 0.42 | KJ414381 |
| TLR3_7 | GGTTATCAGCGCGCC | TLR3_2 | 1 | 0.04 | 0.08 | 0.00 | KJ414382 |
| TLR3_8 | GGTTAGCGGCGCGTC | TLR3_3 | 1 | 0.04 | 0.08 | 0.00 | KJ414383 |
| TLR3_9 | AGTTCGCGGCGCGCC | TLR3_1 | 1 | 0.04 | 0.08 | 0.00 | KJ414384 |
| TLR3_10 | AGTTAGCGACGCGCC | TLR3_4 | 1 | 0.04 | 0.08 | 0.00 | KJ414385 |
| TLR3_11 | AGCTAGCGGCACACC | TLR3_5 | 2 | 0.08 | 0.08 | 0.08 | KJ414386 |
| TLR3_12 | AATTAGCGGCGCGCC | TLR3_6 | 1 | 0.04 | 0.00 | 0.08 | KJ414387 |
| TLR3_13 | AGTTAGCGGCGTGCC | TLR3_1 | 1 | 0.04 | 0.00 | 0.08 | KJ414388 |
| TLR3_14 | AGTAAGCGGCGCGCT | TLR3_1 | 1 | 0.04 | 0.00 | 0.08 | KJ414389 |
| TLR3_15 | AGTAAGTGGCGCGCT | TLR3_7 | 1 | 0.04 | 0.00 | 0.08 | KJ414390 |
| TLR3_16 | AGTTAGCGGCGCACC | TLR3_5 | 1 | 0.04 | 0.00 | 0.08 | KJ414391 |
| TLR3_17 | AGTTAGTGGAGCGCC | TLR3_8 | 1 | 0.04 | 0.00 | 0.08 | KJ414392 |
| TLR4_8 | CGAGGACTCCCCCAGGGGACTC | TLR4_1 | 4 | 0.17 | 0.08 | 0.25 | KJ414393 |
| TLR4_9 | TAACGGCCTCCCCAAGGGACTC | TLR4_2 | 1 | 0.04 | 0.08 | 0.00 | KJ414394 |
| TLR4_10 | CGAGGACTCCTCCAGGGAACTC | TLR4_3 | 1 | 0.04 | 0.08 | 0.00 | KJ414395 |
| TLR4_11 | GGACGACCCCCCCAGGGAGCTC | TLR4_4 | 1 | 0.04 | 0.08 | 0.00 | KJ414396 |
| TLR4_12 | CAACGACTCCCCCAGGGGACTC | TLR4_5 | 1 | 0.04 | 0.08 | 0.00 | KJ414397 |
| TLR4_13 | GAACGACCCCCCCAGGGGACTC | TLR4_5 | 1 | 0.04 | 0.08 | 0.00 | KJ414398 |
| TLR4_14 | TGACGAGTCCCGCAGGGGACTC | TLR4_6 | 2 | 0.08 | 0.17 | 0.00 | KJ414399 |
| TLR4_15 | CAACGACCCCCCCAGGGGACGC | TLR4_5 | 2 | 0.08 | 0.08 | 0.08 | KJ414400 |
| TLR4_16 | CGACGACTCCCCCAGGGAGCTT | TLR4_4 | 1 | 0.04 | 0.08 | 0.00 | KJ414401 |
| TLR4_17 | GGACGACCCCCGCAGGGGACTC | TLR4_5 | 1 | 0.04 | 0.08 | 0.00 | KJ414402 |
| TLR4_18 | TGACGACTCCCGCAGGGGAATC | TLR4_4 | 1 | 0.04 | 0.08 | 0.00 | KJ414403 |
| TLR4_19 | CAACGACTCCCGCAGGGGACTC | TLR4_5 | 2 | 0.08 | 0.00 | 0.17 | KJ414404 |
| TLR4_20 | CGAGGACCCTCCCAGAGAGCTC | TLR4_7 | 1 | 0.04 | 0.00 | 0.08 | KJ414405 |
| TLR4_21 | CGACGACTCCCCCGGGGGACTC | TLR4_8 | 1 | 0.04 | 0.00 | 0.08 | KJ414406 |
| TLR4_22 | TAGCGAGTCCCGCAGGGGACTC | TLR4_9 | 1 | 0.04 | 0.00 | 0.08 | KJ414407 |
| TLR4_23 | CAACAACTCCCCCAGGAGACTC | TLR4_10 | 1 | 0.04 | 0.00 | 0.08 | KJ414408 |
| TLR4_24 | CGACGACTCCCCCAGGGGACTC | TLR4_5 | 1 | 0.04 | 0.00 | 0.08 | KJ414409 |
| TLR4_25 | CGAGGACTCCCCGAGGGAACTC | TLR4_11 | 1 | 0.04 | 0.00 | 0.08 | KJ414410 |
| TLR21_6 | CCCCTG | TLR21_1 | 1 | 0.04 | 0.07 | 0.00 | KJ414411 |
| TLR21_7 | CCTCCG | TLR21_1 | 7 | 0.29 | 0.36 | 0.20 | KJ414412 |
| TLR21_8 | CCCCCG | TLR21_1 | 12 | 0.50 | 0.33 | 0.80 | KJ414413 |
| TLR21_9 | CCCTCG | TLR21_2 | 1 | 0.04 | 0.07 | 0.00 | KJ414414 |
| TLR21_10 | CTCCTG | TLR21_3 | 1 | 0.04 | 0.07 | 0.00 | KJ414415 |
| TLR21_11 | CCCCTA | TLR21_4 | 1 | 0.04 | 0.07 | 0.00 | KJ414416 |
| TLR21_12 | GCCCCG | TLR21_5 | 1 | 0.04 | 0.07 | 0.00 | KJ414417 |
| Total | 62 | 38 |  |  |  |  |  |

^1^Number of individuals with the allele across all populations

^2^Overall frequency in the pooled populations

Table S4. Mean ratio of nonsynonymous to synonymous substitutions (ω, *P* values for hypothesis of purifying selection, dN < dS, in brackets) across identified alleles of the five TLR loci in 1) Berthelot’s pipit (Aber) and 2) both Berthelot’s and tawny pipits (Aber+Acam). Significant values are in bold and underlined.

| Locus | ω (Aber) | ω (Aber+Acam) |
| --- | --- | --- |
| TLR1LA | 0.25 (0.085) | **0.27 (0.010)** |
| TLR1LB | **0.13 (0.018)** | **0.18 (0.002)** |
| TLR3 | 0.50 (0.203) | **0.39 (0.040)** |
| TLR4 | 0.71 (0.389) | **0.24 (0.014)** |
| TLR21 | 0.30 (0.169) | 0.22 (0.056) |

Table S5. Codons in TLR1LA, TLR3 and TLR4 identified as being under positive selection across Berthelot’s and tawny pipits, using two different methods: FUBAR and MEME. Codon numbers correspond to the chicken mRNA for the respective TLR locus. Codons detected by both methods are shown in bold.

| Locus | FUBAR | MEME |
| --- | --- | --- |
| TLR1LA | 392**, 500,** 502 | **500** |
| TLR3 | **260, 261** | 196**,** 207, 216, 224, 238, **260**, **261**, 305, 350, 380, 466 |
| TLR4 | **252**, 332 | **252** |

Figures S1 – S5: Maximum likelihood phylogenetic trees of haplotypes at five TLR loci in five bird species: *Came* = house finch, *Carpodacus mexicanus*; *Peau* = New Zealand robin, *Petroica australis rakiura*; *Fana* = Lesser kestrel, *Falco naumanni*; *Anbe* = Berthelot’s pipit, *Anthus berthelotii,* and *Anca* = tawny pipit, *Anthus campestris.* Figure S1 = TLR1LA, S2 = TLR1LB, S3 = TLR3, S4 = TLR4, S5 = TLR21. Node values represent bootstrap support. Subtrees for *Peau, Anca* and *Fana* were collapsed. Height of the collapsed subtree is proportional to the number of haplotypes in the subtree.

Figures S6 – S10: Networks of TLR haplotypes found in populations of Berthelot’s pipits (Yellow: Canary Islands, Blue: Selvagens, Green: Madeira) and in tawny pipits (white circles). Each circle represents one haplotype. Connections between circles denote the number of nucleotide substitutions needed to change from one haplotype to another. Nonsynonymous substitutions are marked in red. Haplotype number is denoted beside each circle and size of the circle is proportional to the abundance of the haplotype in Berthelot’s pipits. Circles representing tawny pipit haplotypes are drawn at a standard size, and are for comparison of relationships with Berthelot’s pipit haplotypes only.
